# Antibiotics and Bacterial Resistance—A Short Story of an Endless Arms Race

**DOI:** 10.3390/ijms24065777

**Published:** 2023-03-17

**Authors:** Aleksandra Baran, Aleksandra Kwiatkowska, Leszek Potocki

**Affiliations:** 1 Department of Biotechnology, College of Natural Sciences, University of Rzeszów, Pigonia 1, 35-310 Rzeszow, Poland; 2 Institute of Physical Culture Studies, College of Medical Sciences, University of Rzeszów, ul. Towarnickiego 3, 35-959 Rzeszów, Poland

**Keywords:** antibiotic resistance, antibiotics, bacteria, mechanism of action

## Abstract

Despite the undisputed development of medicine, antibiotics still serve as first-choice drugs for patients with infectious disorders. The widespread use of antibiotics results from a wide spectrum of their actions encompassing mechanisms responsible for: the inhibition of bacterial cell wall biosynthesis, the disruption of cell membrane integrity, the suppression of nucleic acids and/or proteins synthesis, as well as disturbances of metabolic processes. However, the widespread availability of antibiotics, accompanied by their overprescription, acts as a double-edged sword, since the overuse and/or misuse of antibiotics leads to a growing number of multidrug-resistant microbes. This, in turn, has recently emerged as a global public health challenge facing both clinicians and their patients. In addition to intrinsic resistance, bacteria can acquire resistance to particular antimicrobial agents through the transfer of genetic material conferring resistance. Amongst the most common bacterial resistance strategies are: drug target site changes, increased cell wall permeability to antibiotics, antibiotic inactivation, and efflux pumps. A better understanding of the interplay between the mechanisms of antibiotic actions and bacterial defense strategies against particular antimicrobial agents is crucial for developing new drugs or drug combinations. Herein, we provide a brief overview of the current nanomedicine-based strategies that aim to improve the efficacy of antibiotics.

## 1. Introduction

The discovery of antibiotics was one of the greatest achievements in medicine of the twentieth century. Their introduction into clinical use reduced morbidity and mortality driven by bacterial infections. From the 1930s to the 1960s, the number of newly identified antibiotics reached its peak; therefore, this period is regarded as the “Golden Age of Antibiotics”. At the same time, strains of antibiotic-resistant bacteria were observed [1]. In the following decades, the overuse and/or misuse of different antimicrobial agents—which has been accelerated by overprescribing antibiotics by clinicians as well as by their widespread use in industry, including animal husbandry and agriculture branches—led to the uncontrolled spread of antibiotic resistance throughout the microbe populations. The most virulent, nosocomial, multidrug-resistant pathogens of clinical importance form a group, which has been referred to as “ESKAPE”. This group includes species such as *Enterococcus faecium*, *Staphylococcus aureus*, *Klebsiella pneumoniae*, *Acinetobacter baumannii, Pseudomonas aeruginosa*, and *Enterobacter* spp., with “ESKAPE” acting as an acronym [2]. Current reports have indicated that a growing number of antibiotic-resistant microorganisms are negatively correlated with the number of effective antibiotics; no new antimicrobial agents have been introduced recently. In light of such data, it is not surprising that the World Health Organization (WHO) has recognized a constantly increasing level of antibiotic resistance, accompanied by a growing number of multidrug-resistant microbes, as a major public health threat of global concern, with the prospect of a return to the “pre-antibiotic era” [3,4]. According to the Centers for Disease Control (CDC) report, antibiotic-resistant microorganisms solely are responsible for two million illnesses in the United States per year; amongst which 23.000 are fatal [5]. Furthermore, it is estimated that ten million people could die from infections caused by antibiotic-resistant bacteria by 2050 [6].

New strategies are being implemented to overcome antimicrobial resistance, some of which focus on the research and development of antibiotics. As a result, since 2017, several new antibiotics have been developed and approved by the U.S. Food and Drug Administration (FDA) and/or the European Medical Agency (EMA) [7,8,9], including cefiderocol, ceftobiprole, rifamycin, eravacycline, sarecycline, omadacycline, plazomicin, contezolid, lefamulin, pretomanid, as well as a combination of imipenem, cilastatin, and relebactam [9]. In addition, dozens of other antimicrobial drugs that act against pathogens on the WHO priority pathogens list are currently under development [7,8,9]. Among the targets of the newly approved antibiotics are mostly the carbapenem-resistant *Enterobacteriaceae* (CRE), oxacillinase-48-producing *Enterobacteriaceae* (OXA-48), and β-lactamase-producing *Enterobacteriaceae* (ESBL) [7,8,9]. This review provides a brief overview of the mechanisms of action of the clinically important antibiotic groups, as well as the mechanisms underlying microbial resistance to particular antimicrobial agents. It is believed that knowledge of these mechanisms should lead to the development of new drugs or their new combinations. This, in turn, may improve the effectiveness of infection therapy caused by bacterial strains resistant to currently available antibiotics. We also briefly present promising nanomedicine-based approaches that aim to improve the efficacy of the old antibiotics, as well as current strategies that focus on searching for new ones.

## 2. Mechanisms of Antibiotic Actions

The term “antibiotic” was introduced by Selman Waksman in 1942 and was defined as a substance produced by microorganisms capable of inhibiting growth or killing other microorganisms [10]. Currently, the definition of antibiotic is broader, and it encompasses compounds of both natural and synthetic origin exhibiting a wide spectrum of actions [11]. The most common mechanisms of antibiotic action include: (i) the inhibition of bacterial cell wall biosynthesis, (ii) the disruption of cell membrane integrity, (iii) the inhibition of nucleic acids and/or protein synthesis, and (iv) disturbances of different metabolic processes [12].

### 2.1. Antibiotics That Inhibit Cell Wall Synthesis

There are two main groups of antibiotics, whose activities lead to the inhibition of cell wall synthesis: β-lactams [13] and glycopeptide antibiotics [14,15,16,17,18]. They achieve this by inhibiting the polymerization of peptidoglycan—which is the primary structural component of the bacterial cell wall [19]—in a direct or indirect manner, respectively [13,14,15,16,17,18]. All β-lactam antibiotics, to which penicillin [20], cephalosporins, carbapenems, and monobactams belong, share a common structure, which is a four-member β-lactam ring (the 3-carbon and 1-nitrogen ring). As the attachment within a lactam is formed between the nitrogen atom and β-carbon atom (relative to carbonyl), this class is known as β-lactams.

**Figure 1:**
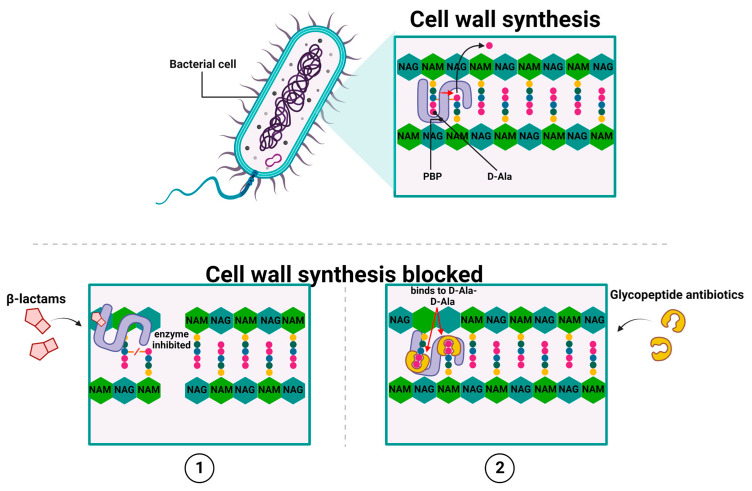
Bactericidal mechanism of (**1**) β-lactams and (**2**) glycopeptide antibiotics; NAG—*N*-acetylglucosamine, NAM—*N*-acetylmuramic acid. Figure created with Biorender.com.

In general, β-lactams act by covalent and irreversible binding to penicillin-binding proteins (PBPs). The PBP exhibit *D, D*-transpeptidases, and *D, D*-carboxypeptidases activities, which are involved in the formation of cross-bridges between neighboring peptide chains of peptidoglycan, thereby blocking peptidoglycan synthesis [21] (Figure 1(1)). 

Differences in chemical structure between the particular representatives of β-lactams affect their relative affinity for different PBPs, which in turn result in discrepancies in their antimicrobial actions, including their activity and spectrum of action. As mentioned above, β-lactam antibiotics have an inhibitory effect on PBPs, enzymes that exhibit *D*, *D*-transpeptidases, and *D*, *D*-carboxypeptidases activities. Specifically, PBP catalyze the final step of peptidoglycan synthesis, which involves the formation of cross-bridges between neighboring peptide chains to ensure cell wall stability [21]. 

The bactericidal activity of β-lactams results from their structural similarity to the peptidoglycan precursor ending in the *D*-alanyl-*D*-alanine group (*D*-Ala-*D*-Ala) [22]. At the molecular level, β-lactam antibiotics target PBP by catalytically acylating an essential serine residue with a reactive β-lactam core. This acylation step yields a stable acyl–enzyme complex (AEC), which is followed by the breaking of the amide bond and the opening of the β-lactam ring [23,24,25]. The inactive acyl-peptidase adduct is characterized by high stability and a long half-life because of its slow hydrolysis. This, in turn, leads to the irreversible inactivation of the enzyme [26] and the inhibition of peptidoglycan biosynthesis, which are altogether followed by the loss of cell wall integrity and, consequently, bacterial cell death [27] (Figure 1(1)). Interestingly, the emergence of new therapeutic options gives reason for optimism in the fight against increasing resistance to beta-lactam antibiotics. These therapeutic allies include the novel beta-lactam-related drugs, i.e., cefiderocol or ceftobiprole. Cefiderocol is a representative of a novel group of antibiotics targeting Gram-negative bacteria, called siderophore cephalosporins, whose mechanism of action consists in preventing cell wall synthesis through binding to PBP proteins (mainly PBP-3) [28]. The first attempts to use siderophores as transporters of antibiotics into the cell were made as early as the 1970s, and this strategy was called the "Trojan horse approach". The unique chemical component of this antibiotic is the addition of a catechol moiety on the C-3 side chain, which chelates iron and mimics naturally occurring siderophore molecules [29]. Siderophores are a structurally diverse group of low-mass molecules (150–2000 Da) with chelating properties and a high affinity for iron. Cefiderocol, after binding iron, reaches the periplasmic space by active transport, which involves, among others, the transporters CirA and Fiu in *E. coli* and PiuA in *P. aeruginosa*. This transport mechanism eliminates the problem of resistance associated with a decrease in the number of porins in the outer membrane or the overexpression of MDR pumps responsible for the efflux phenomenon [28,29]. Moreover, ceftobiprole (BAL 9141) is a new beta-lactam antibiotic that exhibits potent bactericidal activity by binding to PBP, inhibiting transpeptidation, and the formation of the bacterial cell wall, leading to cell lysis and death. The drug can bind to several PBPs found in both Gram-negative and Gram-positive bacteria [30,31]. Ceftobiprole rapidly binds and forms a stable inhibitory acyl–enzyme complex with PBP 2′ (PBP 2a) and PBP 2x, which act on beta-lactam-resistant staphylococci and streptococci, respectively. The stability of the acyl–enzyme complex, combined with the long side chain, which is located deep in the binding pocket of PBP 2′, enhances the binding stability and inhibition of the enzyme [32].

Another highly important group of antibiotics of clinical relevance whose actions lead to the inhibition of cell wall synthesis are glycopeptide antibiotics, such as vancomycin, [14], teicoplanin, oritavancin, and telavancin [15,16]. However, unlike β-lactams, glycopeptide antibiotics inhibit the peptidoglycan biosynthesis in Gram-positive bacteria by targeting lipid II. For example, the mode of action of vancomycin relies on binding to the *D*-Ala-*D*-Ala terminus of the peptidoglycan (PG) cell wall precursor Lipid II (undecaprenyl-diphospho-N-acetylmuramoyl-[N-acetylglucosamine]-l-alanyl-γ-d-glutamyl-l-lysyl-d-alanyl-d-alanine) [33,34]. Such high-affinity binding between them is due to the binding pocket in the vancomycin molecular structure that adopts a conformation corresponding to the spatial structure of *D*-Ala-*D*-Ala. Moreover, an individual antibiotic molecule – through its single carboxyl, single amino, and three amide groups—can form five hydrogen bonds with the corresponding groups of the *D*-Ala-*D*-Ala dipeptide, thus, stabilizing the newly formed complex. This binding blocks the PBP activity and prevents crosslinking Lipid II into mature peptidoglycan, thereby disturbing the maturation process of the peptidoglycan layer [33,34,35,36]. Ultimately, this leads to osmotic shock and bacterial cell death [37,38] (Figure 1(2)).

### 2.2. Antibiotics That Disrupt the Integrity of the Cell Membrane

**Figure 2:**
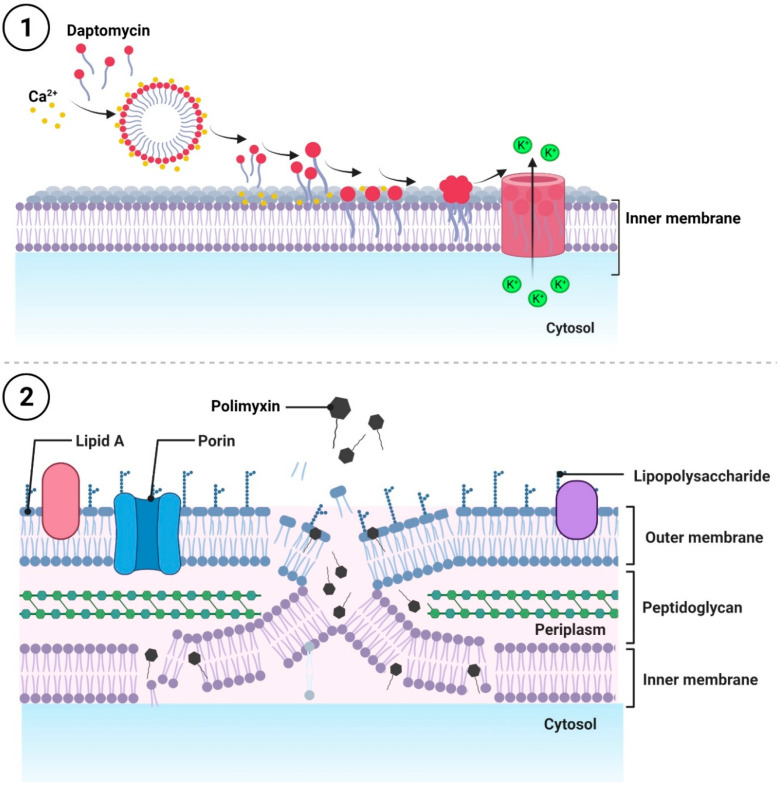
Mechanism of action of (**1**) the lipopeptide antibiotic daptomycin and (**2**) polymyxin. Figure created with Biorender.com.

Amongst the antibiotics whose action leads to the disruption of the bacterial cell membrane, there are: cyclic lipopeptide [39,40,41,42,43,44,45,46,47,48,49] and polymyxins [50,51,52,53,54,55,56,57,58,59,60,61,62,63] (Figure 2). 

The best-known representative of cyclic lipopeptide antibiotics is daptomycin, which was first obtained from the fermentation medium of the soil bacterium *Streptomyces roseosporus* in the 1980s and introduced into clinical practice in 2003 [40,41]. The daptomycin molecule comprises a 13-amino-acid peptide linked to a decanoyl side chain. This linkage depends on the presence of calcium ions [42,43,44], which mask the anionic nature of daptomycin, thereby affecting its physicochemical characteristics. The Ca^2+^–daptomycin complex forms micelles that penetrate the inner membrane, bind to negatively charged phosphatidylglycerol groups, and neutralize them. Then, the Ca^2+^–daptomycin complex is inserted into the membrane and undergoes phosphatidylglycerol-dependent oligomerization. Together, these events lead to the formation of membrane channels, which, combined with the loss of membrane integrity, cause the leakage of ions, mainly potassium and sodium, and a decrease in the transmembrane potential [45,46,47,48,49]. As a result, membrane-related processes are disrupted, eventually causing bacterial cell death (Figure 2(1)). 

Polymyxins are cationic peptide antibiotics produced by the *Paenibacillus polymyxa* subspecies (formerly *Bacillus polymyxa*), after which they are named. There are several polymyxins, such as polymyxin E (also known as colistin), discovered in 1947 [50,51] as well as polymyxins A, B, C, and D. Nevertheless, only polymyxins E and B are clinically useful because of the high toxicity of the others [44]. In general, polymyxins induce chemical instability in the outer cell membrane of Gram-negative bacteria. Due to their amphipathic structure, they act as surface-active compounds. The core of the polymyxin molecule is formed by a cyclic heptapeptide linked to a linear tripeptide side chain. The latter is attached to a branched fatty acid chain of seven to nine carbon atoms via an α-amide bond [53,54,55,56]. The heptapeptide ring consists of amino acids of the *D* and *L* configurations and *L*-α, γ-diaminobutyric acid (Dab). The positively charged residues of α, and γ-diaminobutyric acid react electrostatically with the negatively charged phosphate groups of lipid A, a component of lipopolysaccharide (LPS). This interaction results in the competitive displacement of divalent magnesium and calcium ions, which stabilize the structural arrangement of monolayer LPS molecules. The *N*-terminal hydrophobic lipid chain is then integrated with the lipid regions of the LPS. This leads to an increase in the permeability of the outer membrane, thus facilitating the entry of subsequent antibiotic molecules [56,57,58]. The destructive activity of polymyxins also extends to the inner membrane, resulting in a loss of its physical integrity, a leakage of intracellular components and, consequently, bacterial cell death [58,59,60]. Furthermore, it has been reported that polymyxins can mediate the fusion of the inner leaflet of the outer membrane with the outer leaflet of the inner membrane, promoting the exchange of phospholipids between the membranes. Such changes in the lipid compositions of two membranes cause an osmotic imbalance and cell lysis [61,62,63] (Figure 2(2)).

### 2.3. Antibiotics That Inhibit Nucleic Acid Synthesis

Among the antibiotics that inhibit nucleic acid synthesis as well as being widely used in clinical practice, there are quinolones and rifamycins.

The first discovered antibiotic from the quinolone group was nalidixic acid in 1962, which was obtained as a product of the chloroquine (a drug used in malaria) purification process. Today, nalidixic acid (the first generation of quinolones [64]) is being replaced by a number of its new derivatives with a broad spectrum of bactericidal activity.

Currently, all of them are generally divided into four generations [65]. Quinolones directly affect DNA synthesis and change its topology by targeting two important bacterial enzymes belonging to the type II topoisomerase enzymes: gyrase and topoisomerase IV (Topo IV) [66]. The canonical role of these two enzymes is to regulate the topological state of DNA following both transcription and DNA replication, namely by removing the accumulated positive supercoils. Despite the relatively high similarity between gyrase and topoisomerase IV in their molecular structures, these two enzymes differ from each other in their mechanisms of action. In general, gyrase is responsible for maintaining a steady-state level of negative supercoils in DNA, as well as removing the positive ones ahead of the replication fork or the transcription complex, while topoisomerase IV is engaged in unlinking newly synthesized DNA. In more detail, the heterotetrameric structure of DNA gyrase consists of two GyrA subunits (95 kDa) and two GyrB subunits (90 kDa) (GyrA2:GyrB2). In turn, topoisomerase IV is composed of two ParC subunits (75 kDa) and two ParE subunits (70 kDa) (ParC2:ParE2). Both gyrase and topoisomerase IV share the core type II topoisomerase strand passage mechanisms, which are based on transporting one DNA segment (called the transport or T-segment) through a transient double-strand break in a second segment of DNA (called the gate or G-segment) in an ATP-dependent manner. To prevent newly generated single-stranded DNA ends from being further cleaved, topoisomerases II covalently bind to those 5’-termini, forming the so-called “cleavage complex”. Therefore, the integrity of the genome is preserved during this process. The round of catalysis ends when the transfer strand and products of ATP hydrolysis (since ATP hydrolysis is coupled with the strand passage mechanism) are released and the gate is closed [67,68].

**Figure 3:**
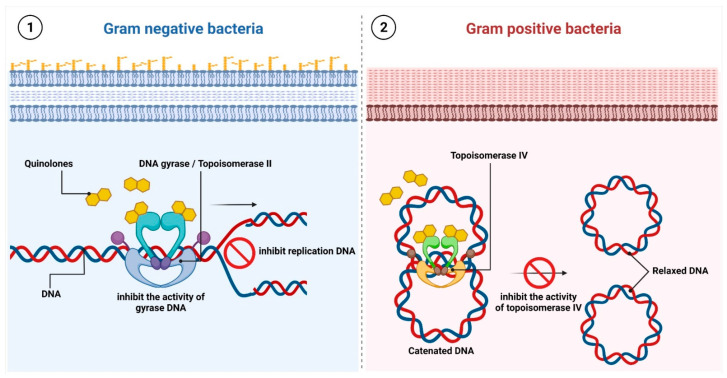
Mechanism of action of quinolones in (**1**) Gram-positive and (**2**) Gram-negative bacteria. Figure created with Biorender.com.

As mentioned above, quinolones inhibit bacterial DNA synthesis by its binding to the topoisomerase–DNA cleavage complex within the G segment. The newly formed quinolone–topoisomerase–DNA complex stabilizes the gap between the nicked DNA strands, thus preventing movement of the replication fork during the ongoing DNA replication process. This results in the inhibition of DNA synthesis and blocks the ability of the bacterial cell to divide, which finally leads to its death [69,70,71,72,73] (Figure 3(1),(2)).

Rifamycins, which belong to the family of naturally derived ansamycin antibiotics, constitute the most important group of antibacterial substances that inhibit the synthesis of bacterial RNA [74,75]. These antibiotics were first isolated in 1959 from the fermentation medium of the *Amycolatopsis mediterranei* strain, as a mixture of several substances that gave rise to rifamycin B and SV [76,77]. Rifamycin SV serves as a precursor of numerous semisynthetic derivatives, including rifampicin, rifabutin, rifapentin, and rifaximin [78]. Importantly, rifamycin was further engineered by Cosmo Technologies Ltd (with use of the Cosmo Pharmaceuticals’ Multi Matrix Technology (MMX®)), in order to allow the colonic release of the active ingredient. In 2018, the drug was approved by the FDA for clinical use in travelers’ diarrhea caused by noninvasive *Escherichia coli* strains [9].

**Figure 4:**
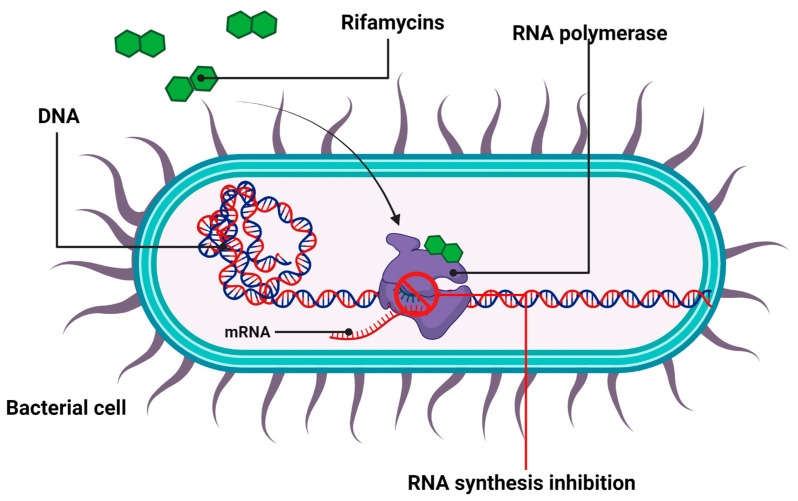
A model of rifamycin action. Figure created with Biorender.com.

A characteristic feature of rifamycins’ structure, which determines their bactericidal properties, is the presence of a macrocyclic ring, which specifically targets the β-subunit of prokaryotic DNA-dependent RNA polymerase (RNAP) near its catalytic center [79]. By such binding, the transcriptional activity of RNAP is inhibited in the transcriptional initiation stage [80], which blocks further extension of the nascent RNA [81,82,83]. This causes a dramatic decrease in protein synthesis followed by bacterial cell death [84] (Figure 4).

### 2.4. Antibiotics That Inhibit Protein Synthesis

A plethora of antibiotics interfere with protein synthesis (which takes place in prokaryotic ribosomes), including tetracyclines, aminoglycosides, macrolides, lincosamides, streptogramins B, and oxazolidinones. Due to the structural differences between the eukaryotic and prokaryotic ribosomes, the antibacterial agents exclusively target the latter and, therefore, are safe for humans [85]. 

**Figure 5:**
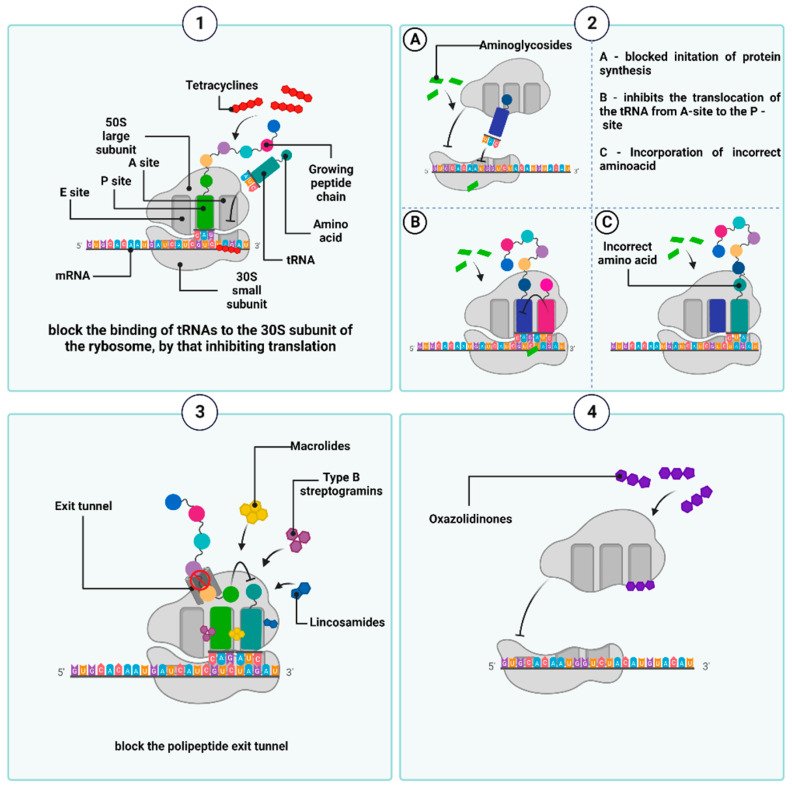
Mechanism of action of (**1**) tetracyclines; (**2**) aminoglycosides; (**3**) MLSB macrolides, lincosamides, and type B streptogramins; (**4**) oxazolidinones. Figure created with Biorender.com.

Tetracyclines are bacteriostatic antibiotics that are currently grouped into three generations. The first one, chlorotetracycline (originally known as aureomycin), was obtained in 1948 from a culture of *Streptomyces aureofaciens* [86,87]. The name ”tetracyclines” is derived from their characteristic chemical structure, which is a core consisting of four flat aromatic hydrocarbon rings (marked as A, B, C, and D) to which various functional groups are attached [88,89]. In an aqueous solution, they exist as hermaphrodite ions, which react with magnesium ions through two oxygen atoms at positions C11 and C12, forming the [Mg (tc)] + complex. This binds to its molecular targets within the 30S subunit of the bacterial ribosome, i.e., the S7 protein and the helix 34 (h34) of the 16S rRNA [75,76,77]. Tetracyclines are also believed to bind to the 50S subunit of the ribosome, albeit to a small extent, and with low specificity [90,91]. As a result of such binding, the aminoacyl-tRNA-transporting amino acid cannot physically bind to acceptor site A within the mRNA–ribosome translation complex, leading to inhibition of the translation. Furthermore, tetracyclines have been shown to compete with tRNA for the P-site of the ribosome, also resulting in the inhibition of polypeptide chain elongation [89,91,92] (Figure 5(1)). 

Importantly, several new-generation tetracycline-class antibiotics have been implemented into clinical practice. In 2018, the fully synthetic fluorocycline (fourth-generation tetracycline antibiotic), eravacycline (TP-434 or 7-fluoro-9-pyrrolidinoacetamido-6-demethyl-6-deoxytetracycline), was approved by both the FDA and EMA, and indicated for the treatment of complicated intra-abdominal infections [7,8,9,93]. Eravacycline acts by binding the bacterial 30S ribosomal subunit, like other tetracyclines. However, due to two unique modifications that are localized at the C-7 (addition of the fluorine atom) and C-9 (addition of the pyrrolidinoacetamo group) positions of its tetracycline core, it overcomes several tetracycline-specific resistance mechanisms acquired by both Gram-positive and Gram-negative bacterial strains (e.g., tetracycline-specific efflux, and ribosomal protection and inactivation). It possesses activity against carbapenem-resistant *Enterobacteriaceae*, methicillin-resistant *Staphylococcus aureus*, ESBL-producing *Enterobacteriaceae*, vancomycin-resistant enterococci, and the majority of anaerobic pathogens [93,94,95]. Additionally, compared to tetracycline, eravacycline binds to the bacterial 30S ribosomal subunit with a ten-fold higher affinity, and a four-fold lower drug concentrations is needed to inhibit protein translation [93,94,95].

Another recently developed and FDA-approved tetracycline-class antibiotic is sarecycline, which has been implemented for the clinical treatment of acne vulgaris. Sarecycline is characterized by the presence of the 7-[methoxy(methyl)amino]methyl] group localized at the C7 position of ring D. In addition to its molecular structure, its mechanism of action also differs to that of other tetracycline antibiotics (but it still exerts its antimicrobial activity by inhibiting protein synthesis). In contrast to other tetracyclines, sarecycline inhibits bacterial ribosomes (partially) by direct mRNA contact, which is mediated by the sarecycline unique C7 moiety (the longest and largest group at this position among all of the tetracyclines). The C7 moiety of sarecycline extends into the messenger RNA (mRNA) channel and directly interacts with the A-site codon. This probably leads to the inhibition of mRNA movement through the channel and its tethering to the 70S ribosome. Another possible consequence of sarecycline action can be a disruption of A-site codon–anticodon interaction, resulting in tRNA accommodation disturbances [96].

Moreover, omadacycline—an aminomethylcycline (a semisynthetic compound derived from tetracycline)—was approved by the FDA in 2018 for use in community-acquired pneumonia and in acute bacterial skin and skin-structure infections. Omadacycline, like other tetracycline-class antibiotics, acts as a protein synthesis inhibitor by binding to the 30S ribosomal subunit. It exhibits broad-spectrum in vitro activity against Gram-positive aerobes, Gram-negative aerobes, anaerobes, and atypical bacteria. Its antimicrobial potency has been increased due to modifications, specifically aminomethyl substitution at the C9 position. It is probably executed by overcoming ribosomal protection (through a higher affinity for the ribosome and/or bypassing protection conferred by ribosomal protection proteins, such as Tet M and Tet O) and efflux-mediated resistance [97,98].

Another group of antimicrobial agents, which can inhibit the synthesis of bacterial protein, are aminoglycosides, which are produced by *Streptomyces* and *Micromonospora* strains and were introduced into routine clinical use nearly eight decades ago [99]. A common feature of their chemical structure is a ring—that is, a streptamine, 2-deoxystreptamine, or streptidine—which is linked by a glycosidic bond to an amino sugar molecule. The different positions of amino and hydroxyl substituents relative to the core structure determine their discrepancies in the specificity of binding to the A-site (and different regions within the A-site) in the 16S rRNA of the 30S subunit of the ribosome. This, in turn, underlies different mechanisms of aminoglycoside actions [100,101,102], although, in general, most of them are responsible for the misreading of mRNA codons and, consequently, the mistranslation. The latter is executed by incorporation of the wrong amino acids into the amino acid sequence of the synthesized peptides. If newly formed, incorrect proteins are incorporated into the cytoplasmic membrane; they deteriorate membrane permeability, affecting transmembrane transport and promoting an increased influx of aminoglycosides into the cell [103]. Some aminoglycosides can inhibit the synthesis of polypeptide chains by suppressing the formation of the initiation complex or blocking the translocation of peptidyl-tRNA from the A-site to the P-site of the ribosome [58,92,104]. Furthermore, the activity of aminoglycosides is also associated with a post-antibiotic effect (PAE), which involves the long-term suppression of bacterial growth despite a decrease in antibiotic concentration below the minimum inhibitory concentration (MIC) value [105] (Figure 5(2)).

Of note, in 2018, the United States Food and Drug Administration approved plazomicin, a next-generation semisynthetic aminoglycoside antibiotic derived from sisomicin, for use in urinary tract infections (cUTI) and pyelonephritis. Plazomicin is a broad-spectrum antibiotic, which is effective against EBSL (extended-spectrum β-lactamase)-producing and carbapenem-resistant *Enterobacteriaceae* (CRE), *Klebsiella pneumoniae* carbapenemase (KPC), as well as gentamycin-resistant strains of *E. coli* strains and colistin (polymyxin B)-producing bacteria [7,8,106,107]. Like other aminoglycoside antibiotics, plazomicin inhibits protein synthesis by binding to the bacterial 30S ribosomal subunit. However, it exhibits a broader spectrum of antibacterial activity due to its unique structure compared to other aminoglycosides. These chemical characteristics include: (i) a lack of hydroxyl groups (which protects against amikacin, gentamicin, and tobramycin resistance); (ii) the presence of an unsaturated hydroxyethyl group; and (iii) N-1 substitution with 4-amino-2-hydroxybutanoic acid (both of these modifications confer protection against aminoglycoside-modifying enzymes, AMEs) [106,107].

The macrolides, lincosamides, streptogramins B, despite the differences in their chemical structures, belong to the same group, known as MLSB, because of their similar mode of action: the inhibition of bacterial protein synthesis by interacting with the 50S ribosomal subunit [108,109].

Macrolides (known since 1952) contain a 14-, 15- or 16-membered lactone ring to which neutral or amino saccharide substituents are attached. Their semisynthetic derivatives of 14-membered-ring representatives, named ketolides, are in turn characterized by a 3-keto group on the lactone ring. 

Unlike macrolides, lincosamides lack a lactone ring; instead, they contain a sugar moiety (α-methylthiolincosamine, α-MTL) and an amino acid moiety (propyl hygric acid). Both of them serve as a structural analogue of the 3′ end of L-Pro-Met-tRNA and deacylated-tRNA interacting with the 23S rRNA of the 50S bacterial ribosomal subunit. The prototype of this class of antibiotics is lincomycin. 

In turn, the streptogramin B molecule features a cyclic hexadepsipeptide. 

MLSB antibiotics exhibit a high affinity for domains II and V of the 23S rRNA molecule next to the nascent polypeptide exit tunnel (NPET) in the large ribosomal subunit (50S). NPET is formed by the L22 and L4 proteins, which build its narrowest constriction. All growing polypeptide chains move throughout the ribosome from the peptidyltransferase (PTC) center (where the synthesis of the peptide bonds takes place) to the NPET, through which they leave the ribosome [110,111]. MLSBs inhibit the peptidyl transferase-dependent formation of a peptide bond between the ester group of peptidyl-tRNA (attached to the P-site of the 50S ribosome) and the nucleophilic amino group of aminoacyl-tRNA (bound to the A-site). Furthermore, the attachment of the antibiotic blocks the entry of NPET before the L4 and L22 ribosomal proteins constrict, inducing the premature dissociation of peptidyl-tRNA from the ribosome (which carries polypeptides of no more than 2 to 10 amino acid residues in length). The accumulation of dissociated peptidyl-tRNA molecules depletes the amount of free tRNA and interrupts protein synthesis [112]. Finally, the MLSBs disrupt the 50S ribosomal subunit formation, as the addition of macrolides to the bacterial culture results in a reduced ratio of 50S to 30S subunit activity of the ribosome [113] (Figure 5(3)).

Oxazolidinones, a group of synthetic antibacterial drugs, date back to the 1980s [114]. Currently, the only oxazolidinones approved for clinical use are linezolid and tedizolid phosphate [115]. It has been suggested that they bind to the pocket at site A of the PTC and act on the aminoacyl tRNA residue also located at this site [116,117,118]. This binding promotes and stabilizes the altered conformation of nucleotide U2585 within the 23S rRNA sequence (which is one of the component of the 50S subunit). When this conserved nucleotide changes its conformation, the peptide bond formation becomes nonproductive, thus negatively affecting protein biosynthesis. For example, in the case of linezolid, this is executed by the formation of a hydrogen bond between the nitrogen atom of U2585 and the oxygen atom of the aromatic C-ring of the antibiotic structure. This leads to conformational changes in the nucleotide U2585 and, consequently, disrupts the correct position of N-formylmethionine-tRNA (fMet-tRNA) at the P-site and blocks the assembly of the 70S translation complex, consisting of the 30S subunit of the ribosome, fMet-tRNA, three initiation factors (IF1, IF2, IF3), GTP, and mRNA [119,120]. If 70S is already formed, the binding of oxazolidinone interrupts protein synthesis by blocking the translocation of the peptide chain from the A- to the P site during the formation of peptide bonds [121] (Figure 5(4)). Another oxazolidinones-group protein synthesis inhibitor that binds to the 50S subunit of bacterial ribosomes, currently in development, is contezolid (MRX-I) [122]. This antibiotic has shown high efficacy in the treatment of bacterial skin and soft tissue infections caused by resistant Gram-positive bacteria. Additionally, contezolid has shown the potential to minimize the limiting adverse effects encountered in linezolid therapy, primarily associated with bone marrow toxicity and serotonergic drug interactions [123].

A new-generation antibiotic, whose action leads to the inhibition of protein synthesis by binding to PTC within the 50S bacterial ribosome subunit (and preventing the binding of transfer RNA for peptide transfer), is lefamulin. It belongs to the pleuromutilin antibiotic class and was approved by the FDA (in 2019) and the European Commission (in 2020) for the treatment of community-acquired bacterial pneumonia (CABP). In general, the pleuromutilin and derivatives are characterized by a tricyclic core, a ketone group, and various C14 side chains, which interact with the A- and P-sites, respectively. This, in turn, leads to the incorrect positioning of tRNA engaged in peptide bond formation [124]. 

An example of an antibiotic with a dual mode of action is pretomanid, i.e, a derivative of nitroimidazoles, which was developed by the non-profit TB Alliance organization [7] and approved by the FDA in 2019 as part of the B-L-Pa regimen (B-L-Pa: bedaquiline, linezolid, pretomanid) for the treatment of tuberculosis. Its antimicrobial activity is aimed against both replicating (in aerobic conditions) and nonreplicating *Mycobacterium tuberculosis* (in anaerobic conditions). In the first case, its activity is executed by inhibiting the oxidation of the hydroxymycolate to ketomycolate blocks cell wall synthesis. In turn, under anaerobic conditions, protomanid acts as a respiratory poison by releasing reactive nitrogen species and, thus, inhibiting *Mycobacterium* protein synthesis [9].

### 2.5. Antimicrobial Substances That Interfere with Metabolic Pathways

Antibiotics can also manifest their antimicrobial effects by disrupting the activity of important metabolic pathways. One of the best-known examples is the inhibition of folic acid synthesis in bacterial cells, mainly either by sulfonamides alone [125] or in combination with trimethoprim due to their synergistic effect [126,127].

**Figure 6:**
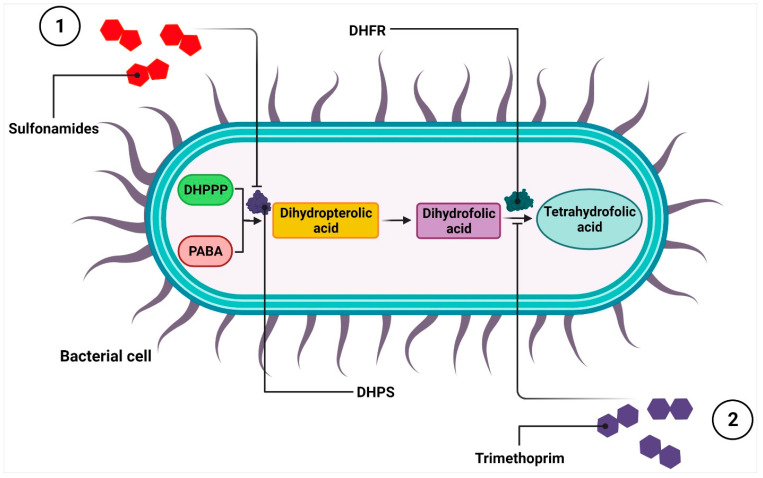
Mechanism of action of antibiotics targeting folic acid synthesis: (**1**) sulfonamides and (**2**) trimethoprim. Dihydropteroate synthase (DHPS), a critical enzyme in the formation of dihydrofolate, is inhibited by sulfamethoxazole, and dihydrofolate reductase (DHFR) is inhibited by trimethoprim. Figure created with Biorender.com.

Sulfonamides belong to the broad-spectrum bacteriostatic antibiotic group [128] encompassing biologically active sulphanilamide [129,130], sulfacetamide, sulfadiazine, sulfamerazine, sulfamethoxazole, sulfanilamide, sulfapyridine, sulfasalazine, sulfathiazole, and sulfisoxazole [131,132]. All of them are structural analogues of para-aminobenzoic acid (PABA), which, in turn, is required for the synthesis of folate by bacteria [126,127,128,129,130,131,132,133]. The antimetabolite activities of sulfonamides are executed by their competition with PABA for the active site of the dihydropteroate synthase (DHPS). The latter catalyzes the condensation of PABA with 7,8-dihydro-6-hydroxymethylpterine pyrophosphate (DHPPP) to dihydropteric acid, which serves as the precursor of dihydrofolic acid. The sulfonamide-dependent inhibition of the of dihydrofolic acid synthesis reduces the amount of metabolically active tetrahydrofolic acid. When folate stores are depleted, the biosynthesis of purines, pyrimidines, and some amino acids required for DNA and protein production is blocked, leading to the inhibition of bacterial growth [126,134,135]. As mammalian cells—unlike bacterial ones—cannot synthesize folate and do not express DHPS, sulfonamide drugs act selectively on bacteria cells drugs [135,136,137,138] (Figure 6(1)). 

In 1962, another chemotherapeutic, called trimethoprim, was introduced as an inhibitor of folic acid synthesis [133,134,135,136,137,138,139,140,141,142]. Through its structural analogy to dihydrofolic acid, it binds to bacterial dihydrofolate reductase (DHFR), which, in turn, converts dihydrofolate to tetrahydrofolate [143]. The inhibition of DHFR and DHPS activities by trimethoprim and sulfonamides, respectively, is another example of synergistic antimicrobial combinations and the only clinically used antifolates to treat bacterial infections [127,141] (Figure 6(2)).

## 3. Mechanisms of Antibiotic Resistance

Bacteria can exhibit two types of antibiotic resistance: intrinsic and acquired. While intrinsic resistance is determined by naturally occurring mechanisms conferred by inherent structural and/or functional features of the bacteria, the acquired resistance results from the changes in the bacterial genome. These consist of mutations in antibiotic-targeted genes or the acquisition of exogenous DNA conferring resistance, horizontally transferred by plasmids, bacteriophages, transposons, or other mobile genetic elements [144,145,146]. 

To date, many independent mechanisms of bacterial resistance to antibiotics have been identified, including primarily modification of the antibiotic target, changes in the cell envelope’s permeability, active pumping of the antibiotic out of the cell (so-called efflux system), and enzymatic inactivation [147].

### 3.1. Modification of the Antibiotic Target Site

The resistance determined by a modification of the target site of an antibacterial substance action constitutes a large and heterogenous group of mechanisms of a different mode of action.

**Figure 7:**
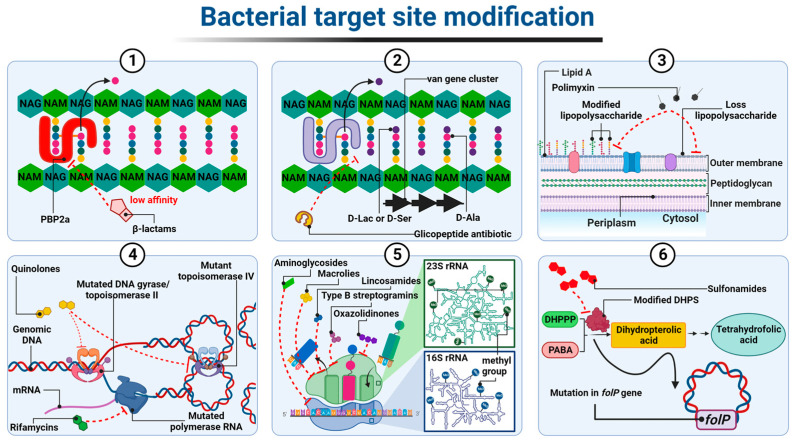
Diagrammatic illustration of some possible resistance mechanisms based on target site modification of antibiotics: (**1**) alteration in PBP; (**2**) altered cell wall precursors; (**3**) modified or loss of lipopolysaccharide; (**4**) mutated DNA gyrase/topoisomerase IV or RNA polymerase; (**5**) alteration in the 30S or 50S subunit; (**6**) modified DHPS. Figure created with Biorender.com.

In the case of β-lactam-resistant bacteria, the resistance is based on a modification of the structure of natural PBP proteins and has been best described in methicillin-insensitive *Staphylococcus aureus* (MRSA) expressing modified PBP2a (also called PBP2′) [148,149]. PBP2a transpeptidases have a reduced affinity for β-lactams but, at the same time, retain catalytic functions [150]. The altered PBPs group also includes PBP1a, PBP2b, and PBP2x enzyme types observed most frequently in *Staphylococcus pneumoniae*, or PBP5 and PBP3r observed in *Enterococcus hirae* S185 isolates [151,152]. The *mecA* and *mecC* genes, which encode PBP2a transpeptidases, determine resistance to almost all β-lactam antibiotics, except cefaroline and ceftobripol. They are located together with the regulatory genes *mecI* and *mecR1* within the staphylococcal SCC*mec* chromosomal cassette integrated into the bacterial chromosome [153,154]. In contrast, the *mecB* gene that determines similar β-lactam antibiotic resistance in *Staphylococcus aureus* was identified in the large plasmid pSAWWU4229_1 [155]. Furthermore, a new *mecD* gene has been detected in *Macrococcus caseolyticus* isolates within so-called genomic resistance island. It has been shown that *mecD* confers resistance to all β-lactam antibiotics, including cephalosporins, ceftaroline, and ceftobiprole [156] (Figure 7(1)).

The resistance to glycopeptides is determined by the synthesis of altered peptidoglycan precursors, which end in carboxyl-terminated *D*-alanyl-*D*-lactate (*D*-Ala-*D*-Lac) or *D*-alanyl-*D*-serine (*D*-Ala-*D*-Ser) The incorporation of the *D*-Lac residue into the Lipid II chain prevents the formation of one out of the five hydrogen bonds that connect Lipid II to the glycopeptide molecule, resulting in an approximately 1000-fold decrease in the affinity of the antibiotic for *D*-Ala-*D*-Lac. In contrast, the substitution of *D*-Ser in place of one *D*-Ala at the precursor end of the pentapeptide leads to an approximately seven-fold decrease in the binding strength of the glycopeptide to *D*-Ala-*D*-Ser, probably due to spherical effects [157,158,159]. The ability to express drug-resistant murein precursors is determined by the presence of Van operons, (i.e., VanA, VanB, VanC, VanD, VanE, VanD, VanG, VanL, VanM, and VanN) in the bacterial genome [160]. It should be noted that the VanA and VanB operons (conferring resistance to mainly vancomycin and teicoplanin or only to vancomycin, respectively) have the highest prevalence in the bacterial world. As they are encoded by genes located in transposons, they can be transferred between different bacterial species using plasmids or chromosome fragments [161,162,163] (Figure 7(2)). 

Resistance to antibiotics that alter the integrity of the outer membrane, i.e., polymyxins, is generally the result of structural modifications of LPS that involve the attachment of positively charged molecules of 4-amino-4-deoxy-*L*-arabinose (L-Ara4N), phosphoethanolamine (PEtN), or galactosamine to the phosphate groups of lipid A [164]. As a result, the negative charge of lipid A is reduced, which leads to the inhibition of the electrostatic interactions of polymyxin molecules with this component. The synthesis of LPS containing L-Ara4N groups is an innate characteristic of *Burkholderia* spp., *Proteus* spp., and *Chromobacterium violaceum* [165,166,167]. In other Gram-negative bacteria, structural modification is mediated by two major two-component regulatory systems, PhoP-PhoQ and PmrA-PmrB. The individual system comprises a sensor histidine kinase, PhoQ or PmrB, and a response regulator, PhoP and PmrA, respectively. The role of the latter is to control gene expression. Both systems have been identified in *Salmonella enterica*, *Klebsiella pneumoniae*, *Pseudomonas aeruginosa*, *Escherichia coli*, and *Yersinia* spp. [168,169,170,171,172]. Under conditions such as low concentrations of Mg^2+^ and Ca^2+^, low pH, or in the presence of polymyxins in the environment, sensor histidine kinase, that is PhoQ or PmrB, trans-autophosphorylates within its dimer. The phosphate is then transferred to response regulators, that is PhoP or PmrA, respectively. The latter positively regulate the transcription of genes responsible for the modification of lipid A in LPS [173].

Furthermore, mutations in genes that encode proteins in these regulatory systems lead to their constitutive activation, which is reflected in the overexpression of genes independently of environmental stimuli and an increased resistance level to polymyxins [174]. 

Of note, *Acinetobacter baumannii* has been confirmed to be the only known LPS-deficient strain to date, due to mutations within the *lpxA*, *lpxC*, and *lpxD* genes, whose products catalyze the initial steps of lipid A biosynthesis [175]. Thus, the polymyxin molecule cannot anchor within the *Acinetobacter baumannii* outer membrane, resulting in cell insensitivity to antibiotics (Figure 7(3)).

The resistance to quinolone antibiotics involves remodeling the chemical structure of gyrase and/or topoisomerase IV, which becomes insensitive to the drug. These structural changes are driven by point mutations in chromosomal genes encoding the gyrase and topoisomerase IV subunits, particularly the GyrA and ParC ones, respectively. They occur within specific codons located in the so-called quinolone resistance determinant region, QRDR within *gyrA*, *gyrB* (encoding gyrase subunits), *parC*, and *parE* (encoding topoisomerase IV subunits) sequences. In the amino acid sequence of the protein, these regions correspond to stretches comprising domains at the amino end of both subunits that are adjacent to the tyrosine residues of their active site [176,177].

There is one exception, however, as serine substitutions have been shown not to affect the efficiency of both topoisomerases II, even in the presence of antibiotics. In addition to the most frequent mutations within *gyrA* and *parC* sequences, mutations that also occur in genes that encode GyrB and ParE subunits of gyrase and topoisomerase IV, respectively, can confer resistance to quinolones [178,179]. Notably, in some species—for example, *Staphylococcus aureus, Streptococcus pneumoniae,* or *Salmonella* spp.—a high level of resistance to quinolones is conferred by several mutations that occur simultaneously in genes encoding both gyrase and topoisomerase IV [180,181,182].

The resistance to rifampicins is related to conformational changes in the β-subunit of DNA-dependent RNA polymerase (RNAP) (resulting in the loss of its affinity for antibiotic) determined by mutations in the *rpoβ* gene (rarely *rpoC* and *rpoA* encoding α subunit of RNAP) [183,184,185,186,187,188,189]. (Figure 7(4)). Among the mechanisms underlying bacterial resistance to antibiotics, which act on the 50S ribosome subunit, are those executed by post-transcriptional modifications of 23S rRNA nucleotides resulting from direct methyltransferase activity. In the case of macrolides (but only those with a 14- or 15-member lactone ring), lincosamides, and streptogramins B, the aforementioned strategy is associated with the presence of methyltransferases encoded by *erm* genes. These enzymes, using *S*-adenosyl-*L*-methionine as a donor methyl group, catalyze the monomethylation or dimethylation reaction of the N6 atom of adenine A2058 (*Escherichia coli* numbering) within the PTC 23S rRNA. Subsequently, this inhibits antibiotic binding to this nucleotide [190,191]. Among the Erm methyltransferases described to date, the ErmC class is the most prevalent in staphylococci (e.g., *Staphylococcus aureus*), while the ErmB and ErmA classes are the most common in enterococci (e.g., *Enterococcus faecalis*) and streptococci (e.g., *Streptococcus pneumoniae*) [192,193,194,195]. *Erm* genes in most microorganisms are located within the transposons and, as a mobile genetic element, they are involved in the spread of the so-called MLSB-type resistance [196,197,198]. In linezolid-resistant bacteria, plasmid-encoded methyltransferases, products of the *cfr* gene, are responsible for the modification of 23S rRNA by adding a methyl group to the C8 atom of its A2503 adenine (*Escherichia coli* numbering). This mechanism, together with the loss of sensitivity to oxazolidinones, leads bacteria to acquire resistance to lincosamides, streptogramins A, macrolides with a 16-member lactone ring, pleuromutilins, and phenicols [199,200]. 

Moreover, the rRNA within the 30S subunit of the ribosome can be methylated in order to protect bacterial cells from bactericidal effect of aminoglycosides. For example, ArmA, a methyltransferase that was primarily identified in *Enterobacteriaceae* and *Acinetobacter baumannii* species, methylates the N7 guanine atom of the G1405 16S rRNA and, consequently, determines high levels of resistance to gentamicin, tobramycin, and amikacin [201].

In addition to rRNA methylation, mutations in genes encoding ribosomal proteins and changes in 16S and 25S rRNA sequences also lead to a decrease in the affinity of protein biosynthesis inhibitors for both ribosomal subunits [202,203]. However, mutations that occur in genes encoding 16S rRNA do not play an important role in bacterial resistance to aminoglycosides, because such molecular changes (with the exception of the A1408G substitution) generally cause bacterial cell death [204,205] (Figure 7(5)).

Modification of the target of an antimicrobial substance is one of the most important mechanisms of the bacterial acquisition of resistance to sulfonamides. Numerous studies have shown that drug-resistant strains produce modified DHPS due to mutations within the conserved regions of the chromosomal *folP* gene (*sulA*). These mutations result in reduced drug sensitivity DHPS. At the same time, drug affinity for PABA is maintained or even increased. In *Neisseria meningitidis* and *Streptococcus pneumoniae* mutants, this type of resistance results from the insertion of six-base-pair-long sequences encoding two additional amino acids in drug-resistant synthetase (Figure 7(6)).

### 3.2. Changes in the Permeability of a Bacterial Cell

The mechanism leading to changes in bacterial cell permeability is commonly employed by Gram-negative bacteria because the structure of their cell wall (when compared to that of Gram-positive bacteria) allows greater regulation of the substance penetration into the cell [206,207,208,209,210,211]. Reduced outer membrane permeability increases bacterial resistance to drugs and is particularly conferred by changes in the qualitative composition of porins, alterations in their functionality or selectivity, as well as in a decrease in porin-encoding gene expression [212].

For example, *Klebsiella pneumoniae* possess two main general diffusion porins, Ompk35 and Ompk36, through which antibiotics, i.e., β-lactams and fluoroquinolones, can enter the cell [213]. The reduced sensitivity of most strains to cefotoxam and cefoxitin, but not carbapenems, is caused by the loss of Ompk35 and Ompk36 porins, which is associated with the simultaneous synthesis of Ompk37 porins with a narrow diffusion channel [214]. On the contrary, the lack of expression of genes encoding both of these porins—which is, generally, driven by point mutations or insertional rearrangements throughout in their coding or promoter sequences—results in the acquisition of resistance to cephalosporins and carbapenems [213,214,215]. 

**Figure 8:**
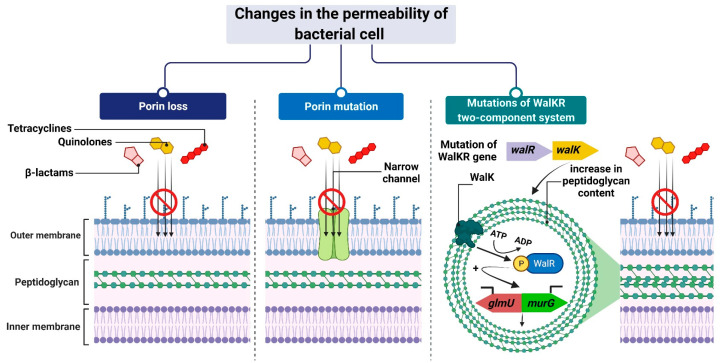
Reduced antibiotic accumulation through changes in the permeability of the bacterial cell. Figure created with Biorender.com.

Another example is the carbapenem-resistant *Pseudomonas aeruginosa* strains, whose resistance to carbapenems (i.e., imipenem and meropenem) is associated with negative transcriptional regulation or mutations in the *oprD* gene that directly inhibits OprD porin synthesis [209,216,217]. Another mechanism is based on a modification of the function of the WalKR two-component regulatory system. This system is characteristic of Gram-positive bacteria with a low content of GC pairs in their genomes. It consists of the histidine kinase WalK and the response regulator WalR, whose orchestrated action alters the expression levels of genes under their control. One such example is genes involved in the regulation of cell wall metabolism [218], such as *glmU* and *murG* [219,220]. Their overexpression, which is driven by mutations in genes that encode components of the regulatory system and/or its upstream elements, results in synthesizing a cell wall with a higher peptidoglycan content [219,220]. The increased thickness of the cell wall makes it more difficult for antibiotic molecules to enter the cell, which contributes to the reduced sensitivity of staphylococci to antibiotics (Figure 8).

### 3.3. Active Pumping of the Antibiotic out of the Cell

One common mechanism of drug resistance is the active efflux of drugs from bac-terial cells to prevent the intracellular accumulation of toxic compounds. Drug-resistant bacteria contain energy-driven drug efflux pumps that squeeze out antibacterial agents, thereby reducing their intracellular concentrations in a way that does not involve alteration or degradation [221]. 

**Figure 9:**
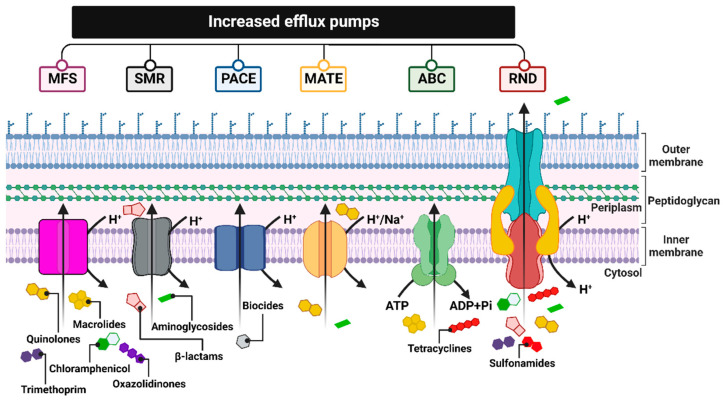
Summary of the six major families of efflux transporters: MFS (a superfamily of the main facilitator), SMR (the small multidrug resistance family), PACE (proteobacterial antimicrobial compound efflux), MATE (multidrug and toxic compound extrusion family), ABC (ATP binding cassette superfamily) and RND (resistance nodulation division family). Figure created with Biorender.com.

These pumps, encoded by genes located on chromosomes, in mobile genetic parts (MGEs) or plasmids, differ in, e.g., their structures, substrate spectrum, and source of energy necessary for transport. Therefore, they are divided into six families: MFS (major facilitator superfamily), SMR (small multidrug resistance family), PACE (proteobacterial antimicrobial compound efflux), MATE (the multidrug and toxic compound extrusion family), ABC (ATP-binding cassette superfamily), and RND (the resistance nodulation division family) [222] (Figure 9).

MSF family proteins are responsible for conferring resistance to fluoroquinolones, macrolides, chloramphenicol, linezolid, trimethoprim, and others. One of the examples is: NorA pumps identified in *Staphylococcus aureus* and MefB observed in *Escherichia coli* strains [223]. 

SMRs, a small multidrug resistance family, are involved in reducing the susceptibility of bacterial cells mainly to β-lactams and some aminoglycosides, as confirmed in *Escherichia coli* (EmeR pump) and *Staphylococcus epidermidis* (SMR pump) isolates [222,224]. 

The PACE pumps identified in the *Acinetobacter baumannii* isolates probably form four trans-membrane α-helices and are composed of 150 amino acids. Their substrate spectrum is limited to commonly used biocides, i.e., chlorohexidine, acriflavine, benzalkonium, or proflavine [222,225,226]. 

MATE-type pumps, acting as antiporters, derive the energy necessary for their activity from the hydrogen or sodium ion gradient. The MATE proteins contribute to the reduced efficacy of fluoroquinolone and some aminoglycoside antibiotics [227]. Among the representatives of this transporter family are: NorM pumps, which are found in *Neisseria gonorrhoeae* [228], and MepA, identified in *Staphylococcus aureus*.

Another family of multidrug efflux pumps is the ATP-binding cassette (ABC) family, which operates through energy derived from the hydrolysis of ATP molecules [229].

ABC pumps, for example, MacB found in *Escherichia coli*, allow bacteria to actively transport antibiotics such as tetracyclines and macrolides outside the cell [230].

Among the different types of efflux pumps, the resistance nodulation division (RND) superfamily is considered the main drug efflux pumps family, as it confers drug resistance to various species of Gram-negative bacteria. 

RND pumps, as well as some ABC, MATE, and MFS pumps, form three-membered protein structures that are located within the entire bacterial cell membrane. The transport proteins of these systems are embedded in the inner membrane (cytoplasmic) and interact with the proteins acting as channels in the outer membrane, as well as acting as the fusion proteins of the periplasmic space (connecting the two membranes), removing antibiotic molecules directly to the external environment. This mechanism of action makes it difficult for the antibiotic to return to the bacterial cell. On the contrary, most MSF and SMR pumps consist of a single protein transporter located in the cytoplasmic membrane, which pumps antibiotic molecules only into the periplasmic space, thus allowing them to easily return to the cytosol. RND pumps, which serve as substrate/H^+^ ion antiport, are characterized by a broad spectrum of transported substrates [231]. The aforementioned pump systems can contribute to multidrug resistance, especially to tetracyclines, chloramphenicol, β-lactams, aminoglycosides, quinolones, sulfonamides, or trimethoprim [232]. Among the RND pump systems identified so far, which, nota bene, are observed only in Gram-negative bacteria, the best known are two ternary complexes: MexAB-OprM in *Pseudomonas aeruginosa* [233] and AcrAB-TolC, which occur in many species of the *Enterobacteriaceae* family, including *Escherichia coli, Salmonella enterica serovar Typhimurium*, or *Klebsiella pneumoniae* [179].

The transport of substances through the efflux system is effectively controlled by local regulatory proteins (e.g., BmrR found in *Bacillus subtilis*), as well as global cellular regulatory proteins (e.g., MarR in *Escherichia coli*) [234]. The overexpression of efflux pumps causes an above-average increase in the efficiency of antibiotic elimination from the bacterial cell and usually results from mutations (deletions, insertions) within the genes encoding these regulatory proteins.

### 3.4. Enzymatic Inactivation

The enzymatic inactivation of antibiotics can be executed by hydrolysis, group transfer, or redox process [235]. In the case of β-lactam antibiotics, resistance is mediated by β-lactamases with hydrolytic enzyme activity, encoded by chromosomal or plasmid genes, which are referred to as abbreviated “*bla*”. They are often a part of mobile genetic elements such as transposons or integrons and, therefore, can be easily transferred between bacteria [236]. These genes can be expressed constitutively or in an inducible β-lactam-dependent way. To date, more than 2000 β-lactamases have been identified. There are two main classifications of these large enzyme groups [237]. Classification according to Ambler, which is based on amino acid sequence homology, divides β-lactamases into four classes, named as A, B, C, and D. On the contrary, an updated version of the Busch–Jakoby functional division distinguishes three (originally four) groups of β-lactam enzymes, which are numbered 1 to 3, depending on their substrate preference and inhibitor action profile [238]. Representatives of classes A, C, and D (Ambler classification) as well as members of groups 1 and 3 (Busch–Jakoby classification) are serine-containing enzymes in the active center; therefore, they are called serine-β-lactamases (SBLs). In turn, class B (Ambler classification) and group 3 (Busch–Jakoby classification) include metallo-β-lactamases (MBLs) with a single Zn^2+^ ion or a pair of Zn^2+^ ions bound to His/Cys/Asp residues in the active center. The hydrolysis reaction of β-lactams catalyzed by SBL proceeds in two steps. After binding to the antibiotic molecule, the serine within the catalytic center attacks the carbonyl group on the β-lactam ring. This results in the hydrolysis of the amide bond of the β-lactam ring and acylation of the enzyme. Then, with the participation of a water molecule, the enzyme is deacylated and the inactive antibiotic with an open Β-lactam ring is released. A different mechanism is observed for MBL. These enzymes use a zinc cation-coordinated hydroxyl group of the water molecule to inactivate the antibiotic [236,237]. Class A enzymes (subgroups 2a, 2b, 2be, 2br, 2ber, 2c, 2e, and 2f) are the most common of all β-lactamases. Enzymes of this class include PC1 penicillinases encoded by the *blaZ* gene, showing a narrow spectrum of activity against penicillins, TEM and SHV type β-lactamases hydrolyzing penicillins and early cephalosporins, *Klebsiella pneumoniae* carbapenemases (KPC) that inactivate carbapenems, as well as extended-spectrum β-lactamases (ESBL), the vast majority of which arise from point mutations altering the hydrolytic preferences of primary TEM (TEM-1, TEM-2) and SHV (SHV-1) [239]. Without counting the enzymes TEM and SHV ESBL, CTX-M, PER, VER, GES, SFO-1, FEC-1, BES-1, BEL-1, TLA-1, and TLA-2 are also found among ESBL [240]. These enzymes have the ability to hydrolyze third-generation cephalosporins known as oxyimino-β-cephalosporins, such as cefotaxime, ceftriaxone, and ceftazidime. Furthermore, they inactivate first- and second-generation cephalosporins and aztreonam, although cephamycins and carbapenems are not their targets. The activity of most representatives of class A β-lactamases, except KPC, is inhibited by clavulanic acid, tazobactam and, to a lesser extent, sulbactam. The class B enzymes (group 3), to which MBLs belong, determine a high level of resistance to penicillins, cephalosporins, and carbapenems, excluding monobactams and aztreonam. Their activity is not inhibited by the previously mentioned β-lactamase inhibitors (clavulanic acid, tazobactam, and sulbactam). However, they are subjected to inhibition by chelating agents such as EDTA, which have a divalent metal ion-binding effect [241]. The substrate spectrum of members of class C (group 1) includes mainly cephalosporins, (except for cefepime, which belongs to the fourth generation cephalosporins), as well as penicillins and monobactams. Like MBLs, β-lactamases of this class do not hydrolyze carbapenems and atreonam (aztreonam). Although they are resistant to β-lactam inhibitors, they can be inhibited by cloxacillin, oxacillin, and aztreonam [242]. Finally, the enzymes of class D (subgroups 2d, 2d, and 2df), to which OXA-type ESBL belong, are characterized by great diversity with regard to their functional properties. As their name suggests, they hydrolyze not only oxacillin but also cloxacillin, carbapenems, penicillins, and to a limited extent, cephalosporins. However, taking into account their inhibitor profile, in most cases their enzymatic activity is not affected by β-lactamase inhibitors [239,243].

Among the novel β-lactamase inhibitors is a relabactam (a diazabicyclooctane beta-lactamase inhibitor), which specifically targets classes A and C of β-lactamases. It was approved by the FDA in 2019 in combination with imipenem (a carbapenem) and cilastatin (a renal dehydropeptidase-I inhibitor) for the treatment of complicated urinary tract infections (UTIs), pyelonephritis, and complicated intra-abdominal infections in adults. The imipenem/cilastatin/relebactam (Recarbrio™) combination exhibits a synergistic effect: (i) imipenem inactivates PBBs and inhibits the cross-linking of peptidoglycan during cell wall synthesis, and its action is protected by (ii) cilastatin, which reduces imipenem renal metabolism, and iii) relebactam, which protects the imipenem from degradation by Ambler classes A and C β-lactamases and *Pseudomonas*-derived cephalosporinas [244].

**Figure 10:**
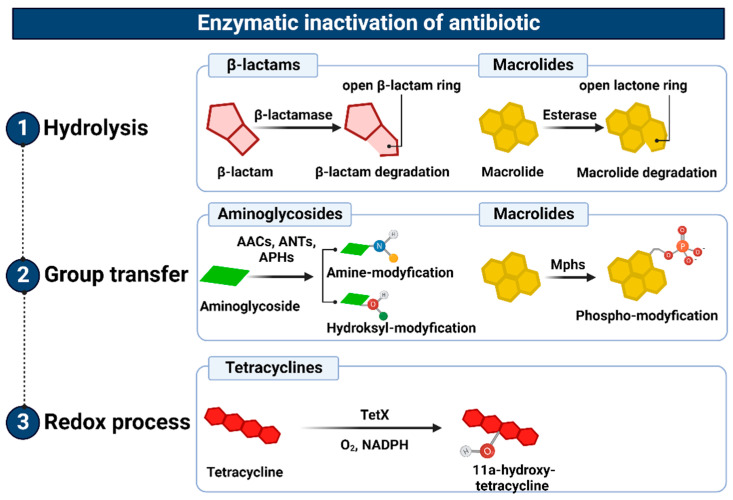
Representation of the enzymatic inactivation of antibiotics through (**1**) hydrolysis, (**2**) group transfer, and (**3**) the redox process. Figure created with Biorender.com.

Similarly, microbial resistance to macrolides can result from enzymatic inactivation of the antibiotic molecule, which is driven by esterases, such as EreA, EreA2, EreB, EreC, and EreD. These enzymes hydrolyze the macrolide lactone ring. Ere esterases are capable of inactivating macrolides with a 14- and 15-member lactone ring, but not those with a 16-member lactone ring. In addition to EreD, which is chromosomally encoded, all other esterases of the Ere family are encoded by genes located in mobile genetic elements. The occurrence of EreA and EreA2 has been described in many pathogenic clinical strains, including non-typhoidal *Salmonella enterica, Pseudomonas* spp., *Vibrio cholera*, and *Klebsiella* spp. EreB, which is distantly related to these esterases, is the most prevalent isolate among all environmental isolates. In the case of EreC, the ereC gene has been identified in the *Enterobacteriaceae* genome [245,246]. In contrast, the presence of the *ereD* gene, which was found in *Riemerella anatipestifer* isolates from ducks, has not yet been found in other bacterial species [247] (Figure 10(1)). 

The inhibition of the antibacterial activity of macrolides with 14-, 15- and 16-membered lactone rings may also be a consequence of their structure modification, involving phosphorylation of the hydroxyl group located in carbon atom C5 of the antibiotic deosamine moiety. So far, 15 macrolide phosphotransferases have been described: MphA, MphB, MphC, MphD, MphE, MphF, MphG, MphH MphI, MPhJ, MphK, MphL, MphM, MphN, MphO. All of them are encoded by genes located on the chromosome or mobile genetic elements. Their occurrence has been confirmed in many bacterial species, both Gram-positive, e.g., *Staphylococcus,* and Gram-negative, e.g., *Escherichia coli* [245].

In addition, the resistance to aminoglycosides depends on phosphotransferase [APH], nucleotidyltransferase [ANT], and acetyltransferase [ACC] activities. The genes encoding these enzymes are located mainly in plasmids, integrons, transposons, or gene cassettes, which together promote their spread throughout bacterial populations [248,249,250,251,252]. Aminoglycoside O-phosphotransferases (APH), which are mainly found among staphylococci and enterococci, catalyze the transfer of a phosphate group from donor ATP (or, in some cases, GTP) to the hydroxyl residue of the aminoglycoside molecule. The APHs are divided into APH(2′), APH(3′), APH(3″), APH(4), APH(6), APH(7″), and APH(9) classes, the most common of which is APH (3′), conferring resistance to kanamycin, neomycin, paromomycin, and others [248,251]. The adenylation of aminoglycosides is another mechanism involved in their inactivation. This, in turn, involves the ATP-dependent transfer of the AMP group to the hydroxyl residue in the aminoglycoside molecule.

The aminoglycoside O-nucleotidyltransferases (ANT) that catalyze this reaction are classified as ANT(2″), ANT(3″), ANT(4′), ANT(6), and ANT(9), the most common of which is ANT(3′′), whose substrate spectrum is limited to streptomycin and spectrinomycin as well as their derivatives. The genes encoding these enzymes have been detected in many Gram-negative bacterial species, including *Escherichia coli*, *Salmonella* spp., *Pseudomonas aeruginosa*, and *Klebsiella pneumoniae* [248,249]. Aminoglycoside N-acetyltransferases (AACs) are the last group of enzymes that confer resistance to aminoglycosides by acetylation of one of the four amino groups (-NH2) in the antibiotic molecule, using acetyl-coenzyme A as a source of acetyl residues. Enzymes of this type include AAC(1), AAC(2), AAC(3′), and AAC(6′) subclasses. These enzymes determine high levels of resistance to gentamicin in several of both Gram-positive and Gram-negative bacterial species [248,249,253], by promoting changes in the aminoglycoside molecule structure and inhibiting its binding to the target site of action, i.e., 16S rRNA of the 30S ribosome subunit (Figure 10(2)).

The above-mentioned mechanisms of antibiotic inactivation through the redox process underlie the resistance of bacteria, e.g., *Sphinogbacterium* spp., to tetracyclines. This inactivation involves a flavin monooxygenase that requires molecular oxygen and NADPH for its activity. This enzyme, encoded by the *tet(X)* gene, catalyzes the hydroxylation of the tetracycline molecule at the position C-11a. The newly formed lla-hydroxytetracycline has a lower magnesium ion coordination capacity than tetracycline and, therefore, does not inhibit protein translation [254,255] (Figure 10(3)).

## 4. At the Dawn of the Post-Antibiotic Era?

It is undisputable that we are facing increasing antibiotic resistance. This is accompanied by a lack of newly discovered and/or developed antibiotics as well as decreases in the effectiveness of the already existing ones [256]. It raises a question: have we already reached the post-antibiotic era? To answer this question, it is necessary to make a brief overview of the alternatives to antibiotics that are currently under development and/or being gradually implemented into clinical practice to combat antimicrobial resistance. Generally, these modern interventions can be divided into strategies, that use naturally occurring phenomena and those that are based on current nanomedicine achievements. 

### 4.1. Natural Born Killers Contra Natural Born Protectors

**Figure 11:**
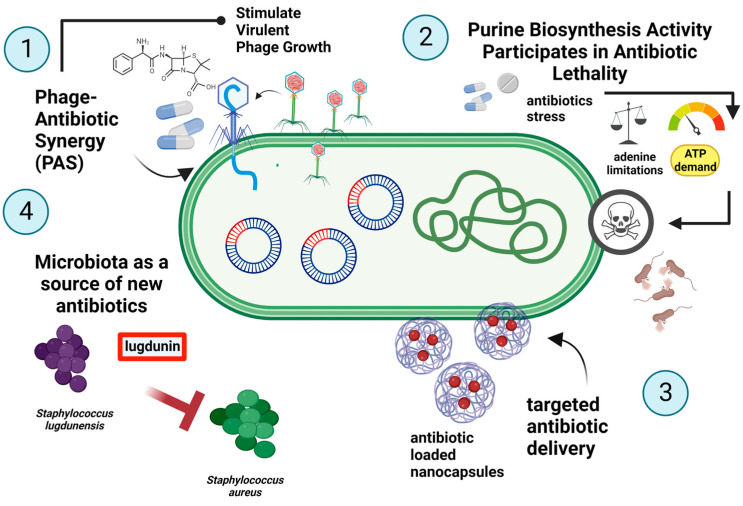
New therapeutic approaches using antibiotics to combat multidrug-resistant bacteria: (**1**) phage-antibiotic synergy (PAS), (**2**) nucleotide metabolism contributes to antibiotic lethality, (**3**) conjugate therapy (nanotechnology + antibiotics), (**4**) microbiota as a source of new antibiotics. Figure created with Biorender.com.

Bacteriophages (phages) are among nature’s solutions that can specifically target and eliminate bacteria. They act as natural killers, infecting specific bacterial hosts by recognizing one or more receptor-binding proteins (RBPs) on the cell surface [257]. Moreover, increasing scientific evidence suggests that phage synergistic interactions with conventional antibiotics have a high therapeutic potential. This bacteriophage–antibiotic synergy (PAS) is defined as a phenomenon in which sublethal concentrations of certain antibiotics can stimulate the replication cycle of lytic phages by the host bacterium [258] (Figure 11(1)). The term was first introduced by Comeau et al. based on a uropathogenic strain of *E. coli* (MFP) and a lytic sifovirus jointly isolated from a patient with a urinary tract infection. This lytic phage (ΦMFP) was found to benefit from sublethal doses of beta-lactams, leading to a larger burst size, and thus, an increase in plaques on agar plates [259,260]. Such combination therapies have been successfully used in the eradication of *Klebsiella pneumoniae* B5055 biofilms or to study the mechanism of PAS in *Escherichia coli*, where the addition of low-dose cefotaxime and cephalosporins led to cell filamentation, and, simultaneously, blocked cell division. This resulted in faster phage assembly during infection, most likely by making larger or altered precursor pools available and/or repairing certain rate-limiting steps in viral replication. Recently, research has emerged on the use of bacteriophages as carriers for the targeted delivery of antibiotics. Targeted drug-carrying phages, first described in 2006, are a powerful tool for the selective elimination of pathogenic bacteria [261,262]. There is currently evidence supporting the utilization of filamentous coliphage f1 molecules in chloramphenicol prodrug conjugation via a hydrophilic linker [262]. It seems that future work will focus on improving targeting, which is crucial for the potency and selectivity of a conjugated drug with a bacteriophage as a carrier. 

On the other hand, there are “natural protectors”, such as human microbiota, whose activity can lead to counteracting the detrimental effects of antimicrobial resistance. A highly intriguing idea is to employ gut microbiota—defined as microorganisms (including bacteria, archaea, viruses, and unicellular eukaryotes) residing in the human gastrointenstinal tract—in order to protect human hosts against antibiotic-resistant pathogens [263,264,265]. In general, this microbiota-mediated protection is executed via the production of antimicrobials against pathogens [266] and/or due to the competitive exclusion of pathogens from their niches [263,265,266]. The approaches aiming to utilize microbiota as a tool against antimicrobial resistance encompass strategies that are based on gut microbiota modulation. Among them are diet and dietary supplements, prebiotics and probiotics, antimicrobial agents, the prophylactic use of antibiotics to decrease the gut occupation by multidrug-resistant organisms, as well as fecal microbiota transplantation [265]. Although these approaches seem to be promising solutions, there is a lack of strong and sufficient evidence in humans so far; in particular, there is a lack of large randomized clinical trials [265]. There are also some other disadvantages; among them there are problems with standardization, as well as putative viral and bacterial infections caused by transplanted pathogens into the host [263,265]. On the one hand, the human gut microbiota serves as a reservoir of resistance genes for at least 50 of 68 classes of antibiotics [267], which is referred to as the gut resistome [265,267,268]. On the other hand, the protective effect of microbiota can be used to predict and minimize the risk of the resistance-gaining recurrence at the individual patient level [269]. Recently, it has been revealed that antibiotic-resistant recurrent and chronic infections are mainly caused by rapid reinfection with the patient’s own bacteria strain/strains, which are resistant to the specific antibiotic. Therefore, the mechanism underlying such types of infections is driven by an unintentional selection for resistant pathogens, rather than de novo acquired resistance. In light of these data, the authors postulate that relying on the individual patient’s past medical history and with the use of the machine learning algorithms, it may be possible to predict and implement personalized antibiotic recommendation. Consequently, it might minimalize the treatment-induced emergence and spread of antibiotic-resistant pathogens [269]. The protective effect of microbiota was also reported by Zipperer et al. (2016), who postulated that a human microbiota should be recognized as a source of new antibiotics. The authors discovered a lugdunin, a novel thiazolidine-containing cyclic peptide antibiotic, which is produced by the human nasal *Staphylococcus lugdunensis* strains. By doing so, *S. lugdunensis* impairs the colonization of pathogenic *S. aureus* and, thus, prevents staphylococcal infections [270] (Figure 11(4)). 

### 4.2. Nanotechnology in the Service of the Antibiotic R&D

Several promising nanotechnology-based approaches aim to enhance the efficacy of the already available antibiotics as well as identify new ones—overall, with an emphasis on strategies against antimicrobial resistance [256,271,272]. 

One of the current groups of strategies is based on “fine-tuning” the old antibiotics by modifying their chemical structure. Such approaches have been successfully implemented for, e.g., cephalosporin [273], tetracycline [274], vancomycin [275,276,277,278], and others [253]. Other approaches encompass strategies that enhance the efficacy of antibiotics through modulation of the bacterial metabolism or improving antibiotics delivery systems [256]. For example, it has been reported that adenine limitation increases the killing effect of antibiotics against *E. coli* by stimulating purine biosynthesis and increasing ATP demand. Together, these increase central carbon metabolism activity and oxygen consumption, leading to enhanced antibiotic lethality [279] (Figure 11(2)).

Due to the achievements of nanomedicine, the efficacy of antibiotics can be enhanced by using so-called “antibiotic nanocarriers”. The role of these nanoparticle carriers—which can be divided into inorganic/organic, carbon-based, and hybrid structures [272]—is to deliver antibiotics directly to their final destinations [256,271] (Figure 11(3)). One of the classes of such nanocarriers is liposomes, which resemble nanocapsules and act as lipid-based surface-functionalized delivery systems protecting antibiotics from degradation. Such a strategy has been employed for the preparation of Arikayce (amikacin liposome inhalation suspension, ALIS), which is an antibiotic–liposome drug approved by the U.S. Food and Drug Administration (FDA). Further evidence for its efficacy was provided by the first real-world study [280]. Arikayce is recommended for the treatment of lung disease caused by a group of bacteria, *Mycobacterium avium* complex (MAC), in patients who do not respond to conventional medical interventions [281]. 

Liposomal encapsulations have also been used by Aradigm Corporation to produce two ciprofloxacin formulations, that is, Lipoquin(^®^) and Pulmaquin(^®^). These inhaled liposomal formulations of ciprofloxacin are dedicated to treating lung infections in cystic fibrosis [282]. 

An attractive alternative to liposomes (which are artificial vesicles) is naive outer membrane vesicles (OMVs). OMVs are composed of natural bacterial surface-exposed proteins in the correct conformation. Antibiotic-loaded OMVs are also used as a delivery system, providing new possibilities for antibiotic development [272,283]. For example, *Acinetobacter baumannii*-derived OMVs loaded with fluoroquinolone antibiotics exhibite good biocompatibility and the ability to kill multidrug-resistant *Pseudomonas aeruginosa*, *Klebsiella pneumoniae*, and enterotoxigenic *Escherichia coli* (ETEC) [284].

The efficacy of antibiotics can also be improved by combining them with nanoparticles (NPs). In general, NPs can interact with bacterial cells, regulate cell membrane penetration, interfere with molecular pathways, as well as enhance the inhibitory effects of antibiotics. For instance, such a synergistic antibacterial effect of NPs with antibiotics has been reported for AgNPs and antibiotics against *Stapylococcus aureus*, *Pseudomonas aeruginosa*, and *Acinetobacter baumannii* [285]. 

With regard to nanomolecules, it is an interesting idea to employ light-activated molecular nanomachines (MNMs). MNMs are synthetic organic nanomolecules possessing a light-induced rotor component. After activation upon light, MNMs drill through the bacterial cell wall as well as disrupt the lipid bilayers of cell membranes with their rapid rotational movement. MNMs also exhibit a synergistic effect when combined with antibiotics. For example, light-activated MNM 1 causes cell wall inner and outer membrane disruptions as well as increased sensitivity of extensively drug-resistant *Klebsiella pneumoniae* to meropenem [286]. 

Another promising idea, which is based on the synergistic effect of nanomaterial-based strategies with antibiotics, is provided by antibacterial photodynamic therapy (aPDT). In general, PDT induces reactive oxygen species (ROS) by using a light-activated photosensitizer [287]. Recently, it was reported that the photosensitizer, that is, 5, 10, 15, 20-Tetrakis(3-hydroxyphenyl)chlorin (temoporfin), suppressed the expression of the antibiotic resistance gene *mecA* (encoding PBP2a) and considerably reduced MRSA drug resistance. The combination of temoporfin with ampicillin or chlorhexidine significantly enhanced the bactericidal effect on MRSA [288]. 

Moreover, innovative strategies have been developed in order to identify new antibiotics; among them is the in silico screening of a small-molecule library. This strategy was successful, for example, in the identification of two PBP2a inhibitors against methicillin-resistant *Staphylococcus aureus* (MRSA). The PBP2a protein is a modified PBP protein produced by MRSA, which determines resistance to β-lactam antibiotic [256]. The newly identified PBP2a inhibitors against MRSA, such as (E)-3-(3-carboxyphenyl)-2-(4-cyanostyryl)quinazolin-4(3H)-one [289] and quinazolinones [290], could help to overcome this problem. 

## 5. Conclusions

Antibiotics are characterized by a wide range of mechanisms of action in bacterial cells. Unfortunately, it is widely known that bacteria have been constantly and relatively quickly acquiring novel resistance mechanisms in response to the newly introduced antibiotics into clinical practice, which is an inevitable process of directed evolution [291]. Furthermore, because a wide range of different strategies has been employed by bacteria, such as horizontal gene transfer, conjugation, transduction processes, and many others, the genetic factors underlying resistance mechanisms can be transferred between different bacterial species, even between those phylogenetically unrelated. It is also widely accepted that the adaptive nature of biological processes will favor the best-fit individuals and promote—in this particular case—the widespread development of antibiotic-resistant strains in the environment. Humans also contribute significantly to such a phenomenon. The increasing consumption of antibiotics, their abuse and/or misuse, as well as human tourist traffic, are among the main factors responsible for the increase in the number of bacterial species with multidrug resistance [292]. 

Taking into account this worldwide problem and in the light of the current achievements in nanomedicine, the following question should be raised: is there still a place for antibiotics in the post-antibiotic world? Moreover, one might consider whether it is still worth debating traditional antibiotics, rather than just their modern substitutes. In fact, the number of new antibiotics reaching the market has considerably decreased in the last 30 years. A lack of diversity and novelty in antibiotic R&D has also been reported throughout the last few decades [293,294,295]. However, this has resulted from economic rather than scientific problems, since for big pharmaceutical companies, other drugs, such as cancer drugs, are much more profitable than antibiotics [295]. At the same time, the need for the development of new antibiotics is still widely discussed and recognized as a problem that should be addressed. Such situations have forced completely novel and unprecedented approaches [293,294,295].

After the rather unsuccessful attempts of replacing Big Pharma by small and medium-sized (SME) companies (since many SME companies have failed due to financial problems or bankruptcy), several new initiatives have appeared. One such solution is the AMR Action Fund, a form of collaboration between pharmaceutical industry and the World Health Organisation (WHO), The European Investment Bank (EIB), and the Wellcome Trust. They intend to invest around USD 1B, as well as to deliver two to four new antibiotics to patients by 2030 [295,296].

The story of antibiotics by no means ends here, since old antibiotics are "fine-tuned” by different chemical modifications, while new ones are being searched for in a wide range of sources, including invertebrates, algae, insects, microbiomes, and others [256,272]. Moreover, innovative strategies are being implemented in order to, e.g., deliver antibiotics to their final destinations more precisely, effectively, and safely.

In conclusion, knowledge of the mechanisms of action of antibiotics is crucial for developing innovative strategies, as it enables the prediction of putative bacterial defense mechanisms; therefore, each particular antimicrobial strategy can be made more effective and precise.

## Data Availability

No new date were created.

## Abbreviations

AAC	aminoglycoside acetyltransferases
ALIS	amikacin liposome inhalation suspension
ANT	nucleotidyltransferases
aPDT	antibacterial photodynamic therapy
APH	aminoglycoside O-phosphotransferase
aPTD	antibacterial photodynamic therapy
DHFR	dihydrofolate reductase
DHPPP	6- hydroxymethyl-7,8-dihydropterin-pyrophosphate
DHPS	dihydropteroate synthase
ESBLs	extended-spectrum β-lactamases
ETEC	enterotoxigenic Escherichia coli
LPS	lipopolysaccharide
MAC	Mycobacterium avium complex
MBLs	metallo-β-lactamases
MLSB group	macrolides, lincosamides, and streptogramines
MNMs	molecular nanomachines
MRSA	methicillin-resistant Staphylococcus aureus
NAG	N-acetylglucosamine
NAM	N-acetylmuramic acid
NPET	nascent polypeptide exit tunnel in the large subunit ribosome
NPs	nanoparticles
OMVs	outer membrane vesicles
PABA	para-aminobenzoic acid
PAS	bacteriophage-antibiotic synergy
PBPs	penicillin-binding proteins
RBPs	receptor-binding proteins
RNAP	DNA-dependent multisubunit RNA polymerase
SBLs	serine β-lactamases

## References

[1] Aslam B., Wang W., Arshad M.I., Khurshid M., Muzammil S., Nisar M.A., Alvi R.F., Aslam M.A., Qamar M.U., Salamat M.K.F. (2018). Antibiotic resistance: A rundown of a global crisis. Infect. Drug Resist..

[2] Gajdács M., Albericio F. (2019). Antibiotic Resistance: From the Bench to Patients. Antibiotics.

[3] World Health Organization (2014). Antimicrobial Resistance: Global Report on Surveillance.

[4] Lack of New Antibiotics Threatens Global Efforts to Contain Drug-Resistant Infections. https://www.who.int/news/item/17-01-2020-lack-of-new-antibiotics-threatens-global-efforts-to-contain-drug-resistant-infections.

[5] (2019). CDC’s Antibiotic Resistance Threats in the United States (2019 AR Threats Report). https://www.cdc.gov/drugresistance/biggest-threats.html.

[6] Antimicrobial Resistance: Tackling a Crisis for the Health and Wealth of Nations/the Review on Antimicrobial Resistance chaired by Jim O’Neill. | Wellcome Collection. https://wellcomecollection.org/works/rdpck35v.

[7] Terreni M., Taccani M., Pregnolato M. (2021). New Antibiotics for Multidrug-Resistant Bacterial Strains: Latest Research Developments and Future Perspectives. Molecules.

[8] Yusuf E., Bax H.I., Verkaik N.J., van Westreenen M. (2021). An Update on Eight “New” Antibiotics against Multidrug-Resistant Gram-Negative Bacteria. J. Clin. Med..

[9] Andrei S., Droc G., Stefan G. (2019). FDA approved antibacterial drugs: 2018-2019. Discoveries.

[10] Clardy J., Fischbach M.A., Currie C.R. (2009). The natural history of antibiotics. Curr. Biol..

[11] Leisner J.J. (2020). The Diverse Search for Synthetic, Semisynthetic and Natural Product Antibiotics From the 1940s and Up to 1960 Exemplified by a Small Pharmaceutical Player. Front. Microbiol..

[12] Uluseker C., Kaster K.M., Thorsen K., Basiry D., Shobana S., Jain M., Kumar G., Kommedal R., Pala-Ozkok I. (2021). A Review on Occurrence and Spread of Antibiotic Resistance in Wastewaters and in Wastewater Treatment Plants: Mechanisms and Perspectives. Front. Microbiol..

[13] Sarkar P., Yarlagadda V., Ghosh C., Haldar J. (2017). A review on cell wall synthesis inhibitors with an emphasis on glycopeptide antibiotics. MedChemComm.

[14] Levine D.P. (2006). Vancomycin: A History. Clin. Infect. Dis..

[15] Binda E., Marinelli F., Marcone G.L. (2014). Old and New Glycopeptide Antibiotics: Action and Resistance. Antibiotics.

[16] Kahne D., Leimkuhler C., Lu A.W., Walsh C. (2005). Glycopeptide and Lipoglycopeptide Antibiotics. Chem. Rev..

[17] Marschall E., Cryle M.J., Tailhades J. (2019). Biological, chemical, and biochemical strategies for modifying glycopeptide antibiotics. J. Biol. Chem..

[18] James R.C., Pierce J.G., Okano A., Xie J., Boger D.L. (2012). Redesign of Glycopeptide Antibiotics: Back to the Future. ACS Chem. Biol..

[19] Scheffers D.-J., Pinho M.G. (2005). Bacterial Cell Wall Synthesis: New Insights from Localization Studies. Microbiol. Mol. Biol. Rev..

[20] Abraham E.P. (1940). An Enzyme from Bacteria able to Destroy Penicillin. Nature.

[21] Wise E.M., Park J.T. (1965). Penicillin: Its basic site of action as an inhibitor of a peptide cross-linking reaction in cell wall mucopeptide synthesis. Proc. Natl. Acad. Sci. USA.

[22] Tipper D.J., Strominger J.L. (1965). Mechanism of action of penicillins: A proposal based on their structural similarity to acyl-D-alanyl-D-alanine. Proc. Natl. Acad. Sci. USA.

[23] Sauvage E., Kerff F., Terrak M., Ayala J.A., Charlier P. (2008). The penicillin-binding proteins: Structure and role in peptidoglycan biosynthesis. FEMS Microbiol. Rev..

[24] Frère J.-M., Duez C., Ghuysen J.-M., Vandekerkhove J. (1976). Occurrence of a serine residue in the penicillin-binding site of the exocellular DD-carboxy-peptidase-transpeptidase fromStreptomycesR61. FEBS Lett..

[25] Massova I., Mobashery S. (1998). Kinship and Diversification of Bacterial Penicillin-Binding Proteins and β-Lactamases. Antimicrob. Agents Chemother..

[26] Edoo Z., Arthur M., Hugonnet J.-E. (2017). Reversible inactivation of a peptidoglycan transpeptidase by a β-lactam antibiotic mediated by β-lactam-ring recyclization in the enzyme active site. Sci. Rep..

[27] Bush K. (2018). Past and present perspectives on β-lactamases. Antimicrob. Agents Chemother..

[28] Krajewska J., Laudy A. (2022). Nowości w lekach przeciwbakteryjnych zarejestrowanych przez europejską agencję leków—odpowiedź na raport who z 2017 r. O globalnym problemie wielolekooporności. Postepy Mikrobiologii.

[29] Sato T., Yamawaki K. (2019). Cefiderocol: Discovery, Chemistry, and In Vivo Profiles of a Novel Siderophore Cephalosporin. Clin. Infect. Dis..

[30] Hebeisen P., Heinze-Krauss I., Angehrn P., Hohl P., Page M.G., Then R.L. (2001). In vitro and in vivo properties of Ro 63-9141, a novel broad-spectrum cephalosporin with activity against methicillin-resistant staphylococci. Antimicrob. Agents Chemother..

[31] Lovering A., Danel F., Page M., Strynadka N. Mechanism of action of ceftobiprole: Structural basis for anti-MRSA activity, Poster P-1586. Proceedings of the 16th European congress of clinical microbiology and infectious diseases (ECCMID).

[32] Kisgen J., Whitney D. (2008). Ceftobiprole, a Broad-Spectrum Cephalosporin With Activity against Methicillin-Resistant Staphylococcus aureus (MRSA). P T.

[33] Allen N.E., LeTourneau D.L., Hobbs J.N. (1997). Molecular interactions of a semisynthetic glycopeptide antibiotic with D-alanyl-D-alanine and D-alanyl-D-lactate residues. Antimicrob. Agents Chemother..

[34] Wang F., Zhou H., Olademehin O.P., Kim S.J., Tao P. (2018). Insights into Key Interactions between Vancomycin and Bacterial Cell Wall Structures. ACS Omega.

[35] Hubbard B.K., Walsh C.T. (2003). Vancomycin Assembly: Nature’s Way. Angew. Chem. Int. Ed..

[36] Zeng D., Debabov D., Hartsell T.L., Cano R.J., Adams S., Schuyler J.A., McMillan R., Pace J.L. (2016). Approved Glycopeptide Antibacterial Drugs: Mechanism of Action and Resistance. Cold Spring Harb. Perspect. Med..

[37] Kövér K.E., Szilágyi L., Batta G., Uhrín D., Jiménez-Barbero J. (2010). Biomolecular Recognition by Oligosaccharides and Glycopeptides: The NMR Point of View. Chem. Biol..

[38] Malin J.J., de Leeuw E. (2019). Therapeutic compounds targeting Lipid II for antibacterial purposes. Infect. Drug Resist..

[39] Epand R.M., Walker C., Epand R.F., Magarvey N.A. (2016). Molecular mechanisms of membrane targeting antibiotics. Biochim. Biophys. Acta (BBA)-Biomembr..

[40] Baltz R.H., Brian P., Miao V., Wrigley S.K. (2005). Combinatorial biosynthesis of lipopeptide antibiotics in Streptomyces roseosporus. J. Ind. Microbiol. Biotechnol..

[41] Lu H., Tonge P.J. (2008). Inhibitors of FabI, an Enzyme Drug Target in the Bacterial Fatty Acid Biosynthesis Pathway. Accounts Chem. Res..

[42] Taylor S.D., Palmer M. (2016). The action mechanism of daptomycin. Bioorganic Med. Chem..

[43] Huang H.W. (2020). DAPTOMYCIN, its membrane-active mechanism vs. that of other antimicrobial peptides. Biochim. Biophys. Acta (BBA)-Biomembr..

[44] Kelesidis T. (2014). The Interplay between Daptomycin and the Immune System. Front. Immunol..

[45] Steenbergen J.N., Alder J., Thorne G.M., Tally F.P. (2005). Daptomycin: A lipopeptide antibiotic for the treatment of serious Gram-positive infections. J. Antimicrob. Chemother..

[46] Baltz R.H. (2009). Daptomycin: Mechanisms of action and resistance, and biosynthetic engineering. Curr. Opin. Chem. Biol..

[47] Silverman J.A., Perlmutter N.G., Shapiro H.M. (2003). Correlation of Daptomycin Bactericidal Activity and Membrane Depolarization in *Staphylococcus aureus*. Antimicrob. Agents Chemother..

[48] Müller A., Wenzel M., Strahl H., Grein F., Saaki T.N.V., Kohl B., Siersma T., Bandow J.E., Sahl H.-G., Schneider T. (2016). Daptomycin inhibits cell envelope synthesis by interfering with fluid membrane microdomains. Proc. Natl. Acad. Sci. USA.

[49] Maget-Dana R., Lakey J.H., Ptak M. (1988). A comparative monomolecular film study of antibiotic A21978C homologues of various lipid chain length. Biochim. Biophys. Acta (BBA) Lipids Lipid Metab..

[50] Velkov T., Roberts K.D., Thompson P.E., Li J. (2016). Polymyxins: A new hope in combating Gram-negative superbugs?. Futur. Med. Chem..

[51] Benedict R.G., Langlykke A.F. (1947). Antibiotic activity of *Bacillus polymyxa*. J. Bacteriol..

[52] Orwa J.A., Govaeris C., Busson R., Roets E., VAN Schepdael A., Hoogmartens J. (2001). Isolation and Structural Characterization of Colistin Components. J. Antibiot..

[53] Komura S., Kurahashi K. (1979). Partial purification and properties of L-2,4-diaminobutyric acid activating enzyme from a polymyxin E producing organism. J. Biochem..

[54] Katz E., Demain A.L. (1977). The peptide antibiotics of Bacillus: Chemistry, biogenesis, and possible functions. Bacteriol. Rev..

[55] Huang E., Yousef A.E. (2014). The lipopeptide antibiotic paenibacterin binds to the bacterial outer membrane and exerts bactericidal activity through cytoplasmic membrane damage. Appl. Environ. Microbiol..

[56] Poirel L., Jayol A., Nordmann P. (2017). Polymyxins: Antibacterial Activity, Susceptibility Testing, and Resistance Mechanisms Encoded by Plasmids or Chromosomes. Clin. Microbiol. Rev..

[57] Storm D.R., Rosenthal K.S., Swanson P.E. (1977). Polymyxin and Related Peptide Antibiotics. Annu. Rev. Biochem..

[58] Yin J., Meng Q., Cheng D., Fu J., Luo Q., Liu Y., Yu Z. (2020). Mechanisms of bactericidal action and resistance of polymyxins for Gram-positive bacteria. Appl. Microbiol. Biotechnol..

[59] Moubareck C.A. (2020). Polymyxins and Bacterial Membranes: A Review of Antibacterial Activity and Mechanisms of Resistance. Membranes.

[60] Velkov T., Thompson P.E., Nation R.L., Li J. (2010). Structure−Activity Relationships of Polymyxin Antibiotics. J. Med. Chem..

[61] Clausell A., Rabanal F., Garcia-Subirats M., Alsina M.A., Cajal Y. (2006). Membrane Association and Contact Formation by a Synthetic Analogue of Polymyxin B and Its Fluorescent Derivatives. J. Phys. Chem. B.

[62] Trimble M.J., Mlynárčik P., Kolář M., Hancock R.E. (2016). Polymyxin: Alternative Mechanisms of Action and Resistance. Cold Spring Harb. Perspect. Med..

[63] Li Z., Velkov T. (2019). Polymyxins: Mode of Action. Adv. Exp. Med. Biol..

[64] Bisacchi G.S. (2015). Origins of the Quinolone Class of Antibacterials: An Expanded “Discovery Story”. J. Med. Chem..

[65] Bush N., Diez-Santos I., Abbott L., Maxwell A. (2020). Quinolones: Mechanism, Lethality and Their Contributions to Antibiotic Resistance. Molecules.

[66] Anderson V., Osheroff V.E.A.A.N. (2001). Type II Topoisomerases as Targets for Quinolone Antibacterials Turning Dr. Jekyll into Mr. Hyde. Curr. Pharm. Des..

[67] Cheng G., Hao H., Dai M., Liu Z., Yuan Z. (2013). Antibacterial action of quinolones: From target to network. Eur. J. Med. Chem..

[68] Champoux J.J. (2001). DNA Topoisomerases: Structure, Function, and Mechanism. Annu. Rev. Biochem..

[69] Correia S., Poeta P., Hébraud M., Capelo J.L., Igrejas G. (2017). Mechanisms of quinolone action and resistance: Where do we stand?. J. Med. Microbiol..

[70] Aldred K.J., Kerns R.J., Osheroff N. (2014). Mechanism of Quinolone Action and Resistance. Biochemistry.

[71] Drlica K., Malik M., Kerns R.J., Zhao X. (2008). Quinolone-Mediated Bacterial Death. Antimicrob. Agents Chemother..

[72] Hawkey P.M. (2003). Mechanisms of quinolone action and microbial response. J. Antimicrob. Chemother..

[73] Pohlhaus J.R., Kreuzer K.N. (2005). Norfloxacin-induced DNA gyrase cleavage complexes block *Escherichia coli* replication forks, causing double-stranded breaks in vivo. Mol. Microbiol..

[74] Phillips I. (1971). Clinical uses and control of rifampicin and clindamycin. J. Clin. Pathol..

[75] Wehrli W. (1977). Ansamycins chemistry, biosynthesis and biological activity. Med. Chem..

[76] Kluepfel D., Lancini G.C., Sartori G. (1965). Metabolism of Barbital by Streptomyces mediterranei. Appl. Microbiol..

[77] Zhao W., Zhong Y., Yuan H., Wang J., Zheng H., Wang Y., Cen X., Xu F., Bai J., Han X. (2010). Complete genome sequence of the rifamycin SV-producing Amycolatopsis mediterranei U32 revealed its genetic characteristics in phylogeny and metabolism. Cell Res..

[78] Rothstein D.M. (2016). Rifamycins, Alone and in Combination. Cold Spring Harb. Perspect. Med..

[79] Ho M.X., Hudson B.P., Das K., Arnold E., Ebright R.H. (2009). Structures of RNA polymerase–antibiotic complexes. Curr. Opin. Struct. Biol..

[80] Floss H.G., Yu T.-W. (2005). RifamycinMode of Action, Resistance, and Biosynthesis. Chem. Rev..

[81] Mosaei H., Molodtsov V., Kepplinger B., Harbottle J., Moon C.W., Jeeves R.E., Ceccaroni L., Shin Y., Morton-Laing S., Marrs E.C.L. (2018). Mode of Action of Kanglemycin A, an Ansamycin Natural Product that Is Active against Rifampicin-Resistant *Mycobacterium tuberculosis*. Mol. Cell.

[82] Kumar K., Chatterji D. (1992). Differential inhibition of abortive transcription initiation at different promoters catalysed byE. coliRNA polymerase Effect of rifampicin on purine or pyramidine-initiated phosphodiester synthesis. FEBS Lett..

[83] Srivastava A., Talaue M., Liu S., Degen D., Ebright R.Y., Sineva E., Chakraborty A., Druzhinin S.Y., Chatterjee S., Mukhopadhyay J. (2011). New target for inhibition of bacterial RNA polymerase: ‘switch region’. Curr. Opin. Microbiol..

[84] Reid P., Speyer J. (1970). Rifampicin Inhibition of Ribonucleic Acid and Protein Synthesis in Normal and Ethylenediaminetetraacetic Acid-Treated *Escherichia coli*. J. Bacteriol..

[85] Arenz S., Wilson D. (2016). Bacterial Protein Synthesis as a Target for Antibiotic Inhibition. Cold Spring Harb. Perspect. Med..

[86] Duggar B.M. (1948). Aureomycin: A product of the continuing search for new antibiotics. Ann. N. Y. Acad. Sci..

[87] Nelson M.L., Levy S.B. (2011). The history of the tetracyclines. Ann. N. Y. Acad. Sci..

[88] Chopra I., Hawkey P.M., Hinton M. (1992). Tetracyclines, molecular and clinical aspects. J. Antimicrob. Chemother..

[89] Chopra I., Roberts M. (2001). Tetracycline Antibiotics: Mode of Action, Applications, Molecular Biology, and Epidemiology of Bacterial Resistance. Microbiol. Mol. Biol. Rev..

[90] Chukwudi C.U. (2016). rRNA Binding Sites and the Molecular Mechanism of Action of the Tetracyclines. Antimicrob. Agents Chemother..

[91] Nguyen F., Starosta A.L., Arenz S., Sohmen D., Dönhöfer A., Wilson D.N. (2014). Tetracycline antibiotics and resistance mechanisms. Biol. Chem..

[92] Giuliodori A.M., Spurio R., Milon P., Fabbretti A. (2019). Antibiotics Targeting the 30S Ribosomal Subunit: A Lesson from Nature to Find and Develop New Drugs. Curr. Top. Med. Chem..

[93] Scott L.J. (2019). Eravacycline: A Review in Complicated Intra-Abdominal Infections. Drugs.

[94] Grossman T.H., Starosta A.L., Fyfe C., O’Brien W., Rothstein D.M., Mikolajka A., Wilson D.N., Sutcliffe J.A. (2012). Target- and resistance-based mechanistic studies with TP-434, a novel fluorocycline antibiotic. Antimicrob. Agents Chemother..

[95] Solomkin J., Evans D., Slepavicius A., Lee P., Marsh A., Tsai L., Sutcliffe J.A., Horn P. (2017). Assessing the Efficacy and Safety of Eravacycline vs Ertapenem in Complicated Intra-abdominal Infections in the Investigating Gram-Negative Infections Treated With Eravacycline (IGNITE 1) Trial: A Randomized Clinical Trial. JAMA Surg..

[96] Batool Z., Lomakin I.B., Polikanov Y.S., Bunick C.G. (2020). Sarecycline interferes with tRNA accommodation and tethers mRNA to the 70S ribosome. Proc. Natl. Acad. Sci. USA.

[97] Durães F., Sousa E. (2019). Omadacycline: A Newly Approved Antibacterial from the Class of Tetracyclines. Pharmaceuticals.

[98] Burgos R.M., Rodvold K.A. (2019). Omadacycline: A novel aminomethylcycline. Infect. Drug Resist..

[99] Durante-Mangoni E., Grammatikos A., Utili R., Falagas M.E. (2009). Do we still need the aminoglycosides?. Int. J. Antimicrob. Agents.

[100] Mingeot-Leclercq M.-P., Glupczynski Y., Tulkens P.M. (1999). Aminoglycosides: Activity and Resistance. Antimicrob. Agents Chemother..

[101] Vakulenko S.B., Mobashery S. (2003). Versatility of Aminoglycosides and Prospects for Their Future. Clin. Microbiol. Rev..

[102] Shakil S., Khan R., Zarrilli R., Khan A.U. (2007). Aminoglycosides versus bacteria—A description of the action, resistance mechanism, and nosocomial battleground. J. Biomed. Sci..

[103] Davis B.D., Chen L.L., Tai P.C. (1986). Misread protein creates membrane channels: An essential step in the bactericidal action of aminoglycosides. Proc. Natl. Acad. Sci. USA.

[104] Wirmer J., Westhof E. (2006). Molecular Contacts Between Antibiotics and the 30S Ribosomal Particle. Methods in Enzymology.

[105] Zhanel G.G., Hoban D.J., Harding G.K. (1991). The Postantibiotic Effect: A Review of in Vitro and in Vivo Data. Dicp.

[106] Alfieri A., Di Franco S., Donatiello V., Maffei V., Fittipaldi C., Fiore M., Coppolino F., Sansone P., Pace M.C., Passavanti M.B. (2022). Plazomicin against Multidrug-Resistant Bacteria: A Scoping Review. Life.

[107] Golkar T., Bassenden A.V., Maiti K., Arya D.P., Schmeing T.M., Berghuis A.M. (2021). Structural basis for plazomicin antibiotic action and resistance. Commun. Biol..

[108] Tsui W.H., Yim G., Wang H.H., McClure J.E., Surette M.G., Davies J. (2004). Dual Effects of MLS Antibiotics: Transcriptional Modulation and Interactions on the Ribosome. Chem. Biol..

[109] Pyörälä S., Baptiste K.E., Catry B., van Duijkeren E., Greko C., Moreno M.A., Pomba M.C.M.F., Rantala M., Ružauskas M., Sanders P. (2014). Macrolides and lincosamides in cattle and pigs: Use and development of antimicrobial resistance. Vet. J..

[110] Douthwaite S., Hansen L.H., Mauvais P. (2000). Macrolide-ketolide inhibition of MLS-resistant ribosomes is improved by alternative drug interaction with domain II of 23S rRNA. Mol. Microbiol..

[111] Eyal Z., Matzov D., Krupkin M., Wekselman I., Paukner S., Zimmerman E., Rozenberg H., Bashan A., Yonath A. (2015). Structural insights into species-specific features of the ribosome from the pathogen *Staphylococcus aureus*. Proc. Natl. Acad. Sci. USA.

[112] Tenson T., Lovmar M., Ehrenberg M. (2003). The Mechanism of Action of Macrolides, Lincosamides and Streptogramin B Reveals the Nascent Peptide Exit Path in the Ribosome. J. Mol. Biol..

[113] Champney W.S., Burdine R. (1995). Macrolide antibiotics inhibit 50S ribosomal subunit assembly in Bacillus subtilis and Staphylococcus aureus. Antimicrob. Agents Chemother..

[114] Slee A.M., Wuonola M.A., McRipley R.J., Zajac I., Zawada M.J., Bartholomew P.T., Gregory W.A., Forbes M. (1987). Oxazolidinones, a new class of synthetic antibacterial agents: In vitro and in vivo activities of DuP 105 and DuP 721. Antimicrob. Agents Chemother..

[115] Rybak J.M., Roberts K. (2015). Tedizolid Phosphate: A Next-Generation Oxazolidinone. Infect. Dis. Ther..

[116] Roger C., Roberts J., Muller L. (2018). Clinical Pharmacokinetics and Pharmacodynamics of Oxazolidinones. Clin. Pharmacokinet..

[117] Dutronc H., Bocquentin F., Galperine T., Lafarie-Castet S., Dupon M. (2005). Le linézolide, premier antibiotique de la famille des oxazolidinones. MÉDecine Et Mal. Infect..

[118] Ippolito J.A., Kanyo Z.F., Wang D., Franceschi F.J., Moore P.B., Steitz T.A., Duffy E.M. (2008). Crystal Structure of the Oxazolidinone Antibiotic Linezolid Bound to the 50S Ribosomal Subunit. J. Med. Chem..

[119] Wilson D.N., Schluenzen F., Harms J.M., Starosta A.L., Connell S.R., Fucini P. (2008). The oxazolidinone antibiotics perturb the ribosomal peptidyl-transferase center and effect tRNA positioning. Proc. Natl. Acad. Sci. USA.

[120] Shaw K.J., Barbachyn M.R. (2011). The oxazolidinones: Past, present, and future. Ann. N. Y. Acad. Sci..

[121] Bozdogan B., Appelbaum P.C. (2004). Oxazolidinones: Activity, mode of action, and mechanism of resistance. Int. J. Antimicrob. Agents.

[122] Wu X., Meng J., Yuan H., Zhong D., Yu J., Cao G., Liu X., Guo B., Chen Y., Li Y. (2021). Pharmacokinetics and Disposition of Contezolid in Humans: Resolution of a Disproportionate Human Metabolite for Clinical Development. Antimicrob. Agents Chemother..

[123] Gordeev M.F., Yuan Z.Y. (2014). New potent antibacterial oxazolidinone (MRX-I) with an improved class safety profile. J. Med. Chem..

[124] Covvey J.R., Guarascio A.J. (2022). Clinical use of lefamulin: A first-in-class semisynthetic pleuromutilin antibiotic. J. Intern. Med..

[125] Sköld O., Mayers D.L. (2009). Sulfonamides and Trimethoprim. Antimicrobial Drug Resistance: Mechanisms of Drug Resistance.

[126] Connor E.E. (1998). Sulfonamide antibiotics. Prim. Care Updat. OB/GYNS.

[127] King C.H., Shlaes D.M., Dul M.J. (1983). Infection caused by thymidine-requiring, trimethoprim-resistant bacteria. J. Clin. Microbiol..

[128] Wood W.B. (1942). Studies on the antibacterial action of the sulfonamide drugs. J. Exp. Med..

[129] Nunes O.C., Manaia C.M., Kolvenbach B.A., Corvini P.F.-X. (2020). Living with sulfonamides: A diverse range of mechanisms observed in bacteria. Appl. Microbiol. Biotechnol..

[130] Gaynes R. (2017). The Discovery of Penicillin—New Insights After More Than 75 Years of Clinical Use. Emerg. Infect. Dis..

[131] Greenwood D., Finch R.G., Greenwood D., Norrby S.R., Whitley R.J. (2010). CHAPTER 29-Sulfonamides. Antibiotic and Chemotherapy.

[132] Giles A., Foushee J., Lantz E., Gumina G. (2019). Sulfonamide Allergies. Pharmacy.

[133] Kalkut G. (1998). Sulfonamides and trimethoprim. Cancer Investig..

[134] Fermér C., Swedberg G. (1997). Adaptation to sulfonamide resistance in Neisseria meningitidis may have required compensatory changes to retain enzyme function: Kinetic analysis of dihydropteroate synthases from N. meningitidis expressed in a knockout mutant of *Escherichia coli*. J. Bacteriol..

[135] Maskell J.P., Sefton A.M., Hall L.M. (1997). Mechanism of sulfonamide resistance in clinical isolates of Streptococcus pneumoniae. Antimicrob. Agents Chemother..

[136] Fermer C., Kristiansen B.-E., Sköld O., Swedberg G. (1995). Sulfonamide resistance in Neisseria meningitidis as defined by site-directed mutagenesis could have its origin in other species. J. Bacteriol..

[137] Achari A., Somers D.O., Champness J.N., Bryant P., Rosemond J., Stammers D.K. (1997). Crystal structure of the anti-bacterial sulfonamide drug target dihydropteroate synthase. Nat. Struct. Biol..

[138] Sköld O. (2000). Sulfonamide resistance: Mechanisms and trends. Drug Resist. Updat..

[139] Eyler R.F., Shvets K. (2019). Clinical Pharmacology of Antibiotics. Clin. J. Am. Soc. Nephrol..

[140] Noall E.W.P., Sewards H.F.G., Waterworth P.M. (1962). Successful Treatment of a Case of Proteus Septicaemia. BMJ.

[141] Huovinen P., Sundström L., Swedberg G., Sköld O. (1995). Trimethoprim and sulfonamide resistance. Antimicrob. Agents Chemother..

[142] Then R.L., Angehrn P. (1979). Low Trimethoprim Susceptibility of Anaerobic Bacteria Due to Insensitive Dihydrofolate Reductases. Antimicrob. Agents Chemother..

[143] Adrian P.V., Klugman K.P. (1997). Mutations in the dihydrofolate reductase gene of trimethoprim-resistant isolates of Streptococcus pneumoniae. Antimicrob. Agents Chemother..

[144] Cox G., Wright G.D. (2013). Intrinsic antibiotic resistance: Mechanisms, origins, challenges and solutions. Int. J. Med. Microbiol..

[145] Woodford N., Ellington M.J. (2007). The emergence of antibiotic resistance by mutation. Clin. Microbiol. Infect..

[146] Gómez-Gómez C., Blanco-Picazo P., Brown-Jaque M., Quirós P., Rodriguez-Rubio L., Cerdà-Cuellar M., Muniesa M. (2019). Infectious phage particles packaging antibiotic resistance genes found in meat products and chicken feces. Sci. Rep..

[147] Peterson E., Kaur P. (2018). Antibiotic Resistance Mechanisms in Bacteria: Relationships Between Resistance Determinants of Antibiotic Producers, Environmental Bacteria, and Clinical Pathogens. Front. Microbiol..

[148] Hawkey P.M. (1998). The origins and molecular basis of antibiotic resistance. BMJ.

[149] Hackbarth C.J., Kocagoz T., Kocagoz S., Chambers H.F. (1995). Point mutations in Staphylococcus aureus PBP 2 gene affect penicillin-binding kinetics and are associated with resistance. Antimicrob. Agents Chemother..

[150] Pinho M.G., de Lencastre H., Tomasz A. (2001). An acquired and a native penicillin-binding protein cooperate in building the cell wall of drug-resistant staphylococci. Proc. Natl. Acad. Sci. USA.

[151] Raze D., Dardenne O., Hallut S., Martinez-Bueno M., Coyette J., Ghuysen J.-M. (1998). The Gene Encoding the Low-Affinity Penicillin-Binding Protein 3r in *Enterococcus hirae* S185R Is Borne on a Plasmid Carrying Other Antibiotic Resistance Determinants. Antimicrob. Agents Chemother..

[152] Harimaya A., Koizumi J.-I., Yamazaki N., Himi T., Yokota S.-I., Sato K., Fujii N. (2006). Alterations of pbp1a, pbp2b, and pbp2x in Streptococcus pneumoniae isolates from children with otolaryngological infectious disease in the Sapporo district of Japan. J. Infect. Chemother..

[153] Matsuhashi M., Song M.D., Ishino F., Wachi M., Doi M., Inoue M., Ubukata K., Yamashita N., Konno M. (1986). Molecular cloning of the gene of a penicillin-binding protein supposed to cause high resistance to beta-lactam antibiotics in Staphylococcus aureus. J. Bacteriol..

[154] Paterson G.K., Harrison E.M., Holmes M.A. (2013). The emergence of mecC methicillin-resistant Staphylococcus aureus. Trends Microbiol..

[155] Becker K., van Alen S., Idelevich E.A., Schleimer N., Seggewiß J., Mellmann A., Kaspar U., Peters G. (2018). Plasmid-Encoded Transferable mecB-Mediated Methicillin Resistance in Staphylococcus aureus. Emerg. Infect. Dis..

[156] Schwendener S., Cotting K., Perreten V. (2017). Novel methicillin resistance gene mecD in clinical Macrococcus caseolyticus strains from bovine and canine sources. Sci. Rep..

[157] Cooper M., Fiorini M.T., Abell C., Williams D.H. (2000). Binding of vancomycin group antibiotics to d -alanine and d -lactate presenting self-assembled monolayers. Bioorganic Med. Chem..

[158] Reynolds P.E., Courvalin P. (2005). Vancomycin Resistance in Enterococci Due to Synthesis of Precursors Terminating in d -Alanyl- d -Serine. Antimicrob. Agents Chemother..

[159] Ge M., Chen Z., Russell H., Onishi H.R., Kohler J., Silver L.L., Kerns R., Fukuzawa S., Thompson C., Kahne D. (1999). Vancomycin Derivatives That Inhibit Peptidoglycan Biosynthesis Without Binding d -Ala- d -Ala. Science.

[160] Ahmed M.O., Baptiste K.E. (2018). Vancomycin-Resistant Enterococci: A Review of Antimicrobial Resistance Mechanisms and Perspectives of Human and Animal Health. Microb. Drug Resist..

[161] Rice L.B. (2012). Mechanisms of Resistance and Clinical Relevance of Resistance to β-Lactams, Glycopeptides, and Fluoroquinolones. Mayo Clin. Proc..

[162] Gagnon S., Lévesque S., Lefebvre B., Bourgault A.-M., Labbé A.-C., Roger M. (2011). vanA-containing Enterococcus faecium susceptible to vancomycin and teicoplanin because of major nucleotide deletions in Tn1546. J. Antimicrob. Chemother..

[163] Hill C.M., Krause K.M., Lewis S.R., Blais J., Benton B.M., Mammen M., Humphrey P.P., Kinana A., Janc J.W. (2010). Specificity of Induction of the *vanA* and *vanB* Operons in Vancomycin-Resistant Enterococci by Telavancin. Antimicrob. Agents Chemother..

[164] Moffatt J.H., Harper M., Boyce J.D. (2019). Mechanisms of Polymyxin Resistance. Polymyxin Antibiotics: From Laboratory Bench to Bedside.

[165] Hamad M.A., Di Lorenzo F., Molinaro A., Valvano M.A. (2012). Aminoarabinose is essential for lipopolysaccharide export and intrinsic antimicrobial peptide resistance in *Burkholderia cenocepacia*. Mol. Microbiol..

[166] Stock I. (2003). Natural Antibiotic Susceptibility of *Proteus* spp., with Special Reference to *P*. *Mirabilis* and *P. penneri* Strains. J. Chemother..

[167] Hase S., Hofstad T., Rietschel E.T. (1977). Chemical structure of the lipid A component of lipopolysaccharides from Fusobacterium nucleatum. J. Bacteriol..

[168] Johnson L., Horsman S.R., Charron-Mazenod L., Turnbull A.L., Mulcahy H., Surette M.G., Lewenza S. (2013). Extracellular DNA-induced antimicrobial peptide resistance in *Salmonella enterica* serovar Typhimurium. BMC Microbiol..

[169] Cheng H.-Y., Chen Y.-F., Peng H.-L. (2010). Molecular characterization of the PhoPQ-PmrD-PmrAB mediated pathway regulating polymyxin B resistance in Klebsiella pneumoniae CG43. J. Biomed. Sci..

[170] Francis V.I., Stevenson E.C., Porter S.L. (2017). Two-component systems required for virulence in *Pseudomonas aeruginosa*. FEMS Microbiol. Lett..

[171] Huang J., Li C., Song J., Velkov T., Wang L., Zhu Y., Li J. (2020). Regulating polymyxin resistance in Gram-negative bacteria: Roles of two-component systems PhoPQ and PmrAB. Futur. Microbiol..

[172] Marceau M., Sebbane F., Ewann F., Collyn F., Lindner B., Campos M.A., Bengoechea J.-A., Simonet M. (2004). The pmrF polymyxin-resistance operon of Yersinia pseudotuberculosis is upregulated by the PhoP-PhoQ two-component system but not by PmrA-PmrB, and is not required for virulence. Microbiology.

[173] Gooderham W.J., Hancock R.E.W. (2009). Regulation of virulence and antibiotic resistance by two-component regulatory systems in *Pseudomonas aeruginosa*. FEMS Microbiol. Rev..

[174] Olaitan A., Morand S., Rolain J.-M. (2014). Mechanisms of polymyxin resistance: Acquired and intrinsic resistance in bacteria. Front. Microbiol..

[175] Moffatt J.H., Harper M., Harrison P., Hale J.D., Vinogradov E., Seemann T., Henry R., Crane B., St. Michael F., Cox A.D. (2010). Colistin Resistance in Acinetobacter baumannii Is Mediated by Complete Loss of Lipopolysaccharide Production. Antimicrob. Agents Chemother..

[176] Hooper D.C., Jacoby G.A. (2015). Mechanisms of drug resistance: Quinolone resistance. Ann. N. Y. Acad. Sci..

[177] Jacoby G.A. (2005). Mechanisms of Resistance to Quinolones. Clin. Infect. Dis..

[178] Hooper D.C., Jacoby G.A. (2016). Topoisomerase Inhibitors: Fluoroquinolone Mechanisms of Action and Resistance. Cold Spring Harb. Perspect. Med..

[179] Ruiz J. (2003). Mechanisms of resistance to quinolones: Target alterations, decreased accumulation and DNA gyrase protection. J. Antimicrob. Chemother..

[180] Wasyl D., Hoszowski A., Zając M. (2014). Prevalence and characterisation of quinolone resistance mechanisms in *Salmonella* spp.. Vet. Microbiol..

[181] Eliopoulos G.M. (2004). Quinolone Resistance Mechanisms in Pneumococci. Clin. Infect. Dis..

[182] Schmitz F.J., Hofmann B., Hansen B., Scheuring S., Lückefahr M., Klootwijk M., Verhoef J., Fluit A., Heinz H.P., Köhrer K. (1998). Relationship between ciprofloxacin, ofloxacin, levofloxacin, sparfloxacin and moxifloxacin (BAY 12-8039) MICs and mutations in grlA, grlB, gyrA and gyrB in 116 unrelated clinical isolates of Staphylococcus aureus. J. Antimicrob. Chemother..

[183] Khan A.S., Phelan J.E., Khan M.T., Ali S., Qasim M., Napier G., Campino S., Ahmad S., Cabral-Marques O., Zhang S. (2021). Characterization of rifampicin-resistant *Mycobacterium tuberculosis* in Khyber Pakhtunkhwa, Pakistan. Sci. Rep..

[184] Campbell E.A., Korzheva N., Mustaev A., Murakami K., Nair S., Goldfarb A., Darst S.A. (2001). Structural Mechanism for Rifampicin Inhibition of Bacterial RNA Polymerase. Cell.

[185] Koch A., Mizrahi V., Warner D.F. (2014). The impact of drug resistance on Mycobacterium tuberculosis physiology: What can we learn from rifampicin?. Emerg. Microbes Infect..

[186] Zaw M.T., Emran N.A., Lin Z. (2018). Mutations inside rifampicin-resistance determining region of rpoB gene associated with rifampicin-resistance in *Mycobacterium tuberculosis*. J. Infect. Public Health.

[187] Padayachee T., Klugman K.P. (1999). Molecular Basis of Rifampin Resistance in *Streptococcus pneumoniae*. Antimicrob. Agents Chemother..

[188] Goldstein B.P. (2014). Resistance to rifampicin: A review. J. Antibiot..

[189] Lin Y.-H., Tai C.-H., Li C.-R., Lin C.-F., Shi Z.-Y. (2013). Resistance profiles and rpoB gene mutations of *Mycobacterium tuberculosis* isolates in Taiwan. J. Microbiol. Immunol. Infect..

[190] Weisblum B. (1995). Erythromycin resistance by ribosome modification. Antimicrob. Agents Chemother..

[191] Schroeder M.R., Stephens D.S. (2016). Macrolide Resistance in Streptococcus pneumoniae. Front. Cell. Infect. Microbiol..

[192] Leclercq R. (2002). Mechanisms of Resistance to Macrolides and Lincosamides: Nature of the Resistance Elements and Their Clinical Implications. Clin. Infect. Dis..

[193] Daoud Z., Kourani M., Saab R., Nader M.A., Hajjar M. (2011). Resistance of Streptococcus pneumoniae isolated from Lebanese patients between 2005 and 2009. Rev. Esp. Quimioter. Publ. Soc. Esp. Quimioter..

[194] Portillo A., Ruiz-Larrea F., Zarazaga M., Alonso A., Martinez J.L., Torres C. (2000). Macrolide Resistance Genes in *Enterococcus* spp.. Antimicrob. Agents Chemother..

[195] Westh H., Hougaard D.M., Vuust J., Rosdahl V.T. (1995). erm genes in erythromycin-resistant Staphylococcus aureus and coagulase-negative staphylococci. APMIS Acta Pathol. Microbiol. Immunol. Scand..

[196] De Leener E., Martel A., Decostere A., Haesebrouck F. (2004). Distribution of the *erm*(B) Gene, *tet* racycline Resistance Genes, and Tn *1545*-like Transposons in Macrolide- and Lincosamide-Resistant Enterococci from Pigs and Humans. Microb. Drug Resist..

[197] Huber L., Giguère S., Slovis N.M., Álvarez-Narváez S., Hart K.A., Greiter M., Morris E.R.A., Cohen N.D. (2020). The novel and transferable *erm* (51) gene confers macrolides, lincosamides and streptogramins B (MLS_B_) resistance to clonal *Rhodococcus equi* in the environment. Environ. Microbiol..

[198] Wipf J.R.K., Schwendener S., Perreten V. (2014). The Novel Macrolide-Lincosamide-Streptogramin B Resistance Gene *erm* (44) Is Associated with a Prophage in Staphylococcus xylosus. Antimicrob. Agents Chemother..

[199] Chen H., Wang X., Yin Y., Li S., Zhang Y., Wang Q., Wang H. (2019). Molecular characteristics of oxazolidinone resistance in enterococci from a multicenter study in China. BMC Microbiol..

[200] Long K.S., Poehlsgaard J., Kehrenberg C., Schwarz S., Vester B. (2006). The Cfr rRNA Methyltransferase Confers Resistance to Phenicols, Lincosamides, Oxazolidinones, Pleuromutilins, and Streptogramin A Antibiotics. Antimicrob. Agents Chemother..

[201] Doi Y., Wachino J.-I., Arakawa Y. (2016). Aminoglycoside Resistance. Infect. Dis. Clin. N. Am..

[202] Zaman S., Fitzpatrick M., Lindahl L., Zengel J. (2007). Novel mutations in ribosomal proteins L4 and L22 that confer erythromycin resistance in *Escherichia coli*. Mol. Microbiol..

[203] Bilgin N., Richter A.A., Ehrenberg M., Dahlberg A.E., Kurland C.G. (1990). Ribosomal RNA and protein mutants resistant to spectinomycin. EMBO J..

[204] Kondo J. (2011). A Structural Basis for the Antibiotic Resistance Conferred by an A1408G Mutation in 16S rRNA and for the Antiprotozoal Activity of Aminoglycosides. Angew. Chem. Int. Ed..

[205] Garneau-Tsodikova S., Labby K.J. (2015). Mechanisms of resistance to aminoglycoside antibiotics: Overview and perspectives. MedChemComm.

[206] Delcour A.H. (2009). Outer membrane permeability and antibiotic resistance. Biochim. Biophys. Acta (BBA) Proteins Proteom..

[207] Yarlagadda V., Manjunath G.B., Sarkar P., Akkapeddi P., Paramanandham K., Shome B.R., Ravikumar R., Haldar J. (2015). Glycopeptide Antibiotic To Overcome the Intrinsic Resistance of Gram-Negative Bacteria. ACS Infect. Dis..

[208] Konovalova A., Kahne D.E., Silhavy T.J. (2017). Outer Membrane Biogenesis. Annu. Rev. Microbiol..

[209] Papp-Wallace K.M., Endimiani A., Taracila M.A., Bonomo R.A. (2011). Carbapenems: Past, Present, and Future. Antimicrob. Agents Chemother..

[210] Pagès J.-M., James C.E., Winterhalter M. (2008). The porin and the permeating antibiotic: A selective diffusion barrier in Gram-negative bacteria. Nat. Rev. Microbiol..

[211] Nikaido H. (2003). Molecular Basis of Bacterial Outer Membrane Permeability Revisited. Microbiol. Mol. Biol. Rev..

[212] Kumar A., Schweizer H.P. (2005). Bacterial resistance to antibiotics: Active efflux and reduced uptake. Adv. Drug Deliv. Rev..

[213] Khalid A., Lubián A.F., Ma L., Lin R.C., Iredell J.R. (2020). Characterizing the role of porin mutations in susceptibility of beta lactamase producing Klebsiella pneumoniae isolates to ceftaroline and ceftaroline-avibactam. Int. J. Infect. Dis..

[214] Doménech-Sánchez A., Hernández-Allés S., Martínez-Martínez L., Benedí V.J., Albertí S. (1999). Identification and Characterization of a New Porin Gene of *Klebsiella pneumoniae*: Its Role in β-Lactam Antibiotic Resistance. J. Bacteriol..

[215] Khalifa S.M., El-Aziz A.M.A., Hassan R., Abdelmegeed E.S. (2021). β-lactam resistance associated with β-lactamase production and porin alteration in clinical isolates of E. coli and K. pneumoniae. PLoS ONE.

[216] El Amin N., Giske C.G., Jalal S., Keijser B., Kronvall G., Wretlind B. (2005). Carbapenem resistance mechanisms in *Pseudomonas aeruginosa*: Alterations of porin OprD and efflux proteins do not fully explain resistance patterns observed in clinical isolates. Apmis.

[217] Studemeister A.E., Quinn J.P. (1988). Selective imipenem resistance in *Pseudomonas aeruginosa* associated with diminished outer membrane permeability. Antimicrob. Agents Chemother..

[218] Takada H., Yoshikawa H. (2018). Essentiality and function of WalK/WalR two-component system: The past, present, and future of research. Biosci. Biotechnol. Biochem..

[219] Peng H., Hu Q., Shang W., Yuan J., Zhang X., Liu H., Zheng Y., Hu Z., Yang Y., Tan L. (2016). WalK(S221P), a naturally occurring mutation, confers vancomycin resistance in VISA strain XN108. J. Antimicrob. Chemother..

[220] Howden B.P., McEvoy C.R.E., Allen D.L., Chua K., Gao W., Harrison P., Bell J., Coombs G., Bennett-Wood V., Porter J.L. (2011). Evolution of Multidrug Resistance during Staphylococcus aureus Infection Involves Mutation of the Essential Two Component Regulator WalKR. PLoS Pathog..

[221] Fernández L., Hancock R.E.W. (2013). Adaptive and Mutational Resistance: Role of Porins and Efflux Pumps in Drug Resistance. Clin. Microbiol. Rev..

[222] Kornelsen V., Kumar A. (2021). Update on Multidrug Resistance Efflux Pumps in *Acinetobacter* spp.. Antimicrob. Agents Chemother..

[223] Aron Z., Opperman T.J. (2018). The hydrophobic trap-the Achilles heel of RND efflux pumps. Res. Microbiol..

[224] Srinivasan V.B., Rajamohan G., Gebreyes W.A. (2009). Role of AbeS, a Novel Efflux Pump of the SMR Family of Transporters, in Resistance to Antimicrobial Agents in *Acinetobacter baumannii*. Antimicrob. Agents Chemother..

[225] Hassan K.A., Liu Q., Henderson P.J.F., Paulsen I.T. (2015). Homologs of the Acinetobacter baumannii AceI Transporter Represent a New Family of Bacterial Multidrug Efflux Systems. Mbio.

[226] Bolla J.R., Howes A.C., Fiorentino F., Robinson C.V. (2020). Assembly and regulation of the chlorhexidine-specific efflux pump AceI. Proc. Natl. Acad. Sci. USA.

[227] Kabra R., Chauhan N., Kumar A., Ingale P., Singh S. (2018). Efflux pumps and antimicrobial resistance: Paradoxical components in systems genomics. Prog. Biophys. Mol. Biol..

[228] Rouquette-Loughlin C., Dunham S.A., Kuhn M., Balthazar J.T., Shafer W.M. (2003). The NorM Efflux Pump of *Neisseria gonorrhoeae* and *Neisseria meningitidis* Recognizes Antimicrobial Cationic Compounds. J. Bacteriol..

[229] Hürlimann L.M., Hohl M., Seeger M.A. (2017). Split tasks of asymmetric nucleotide-binding sites in the heterodimeric ABC exporter EfrCD. FEBS J..

[230] Greene N.P., Kaplan E., Crow A., Koronakis V. (2018). Antibiotic Resistance Mediated by the MacB ABC Transporter Family: A Structural and Functional Perspective. Front. Microbiol..

[231] Li X.-Z., Plésiat P., Nikaido H. (2015). The Challenge of Efflux-Mediated Antibiotic Resistance in Gram-Negative Bacteria. Clin. Microbiol. Rev..

[232] Reygaert W.C. (2018). An overview of the antimicrobial resistance mechanisms of bacteria. AIMS Microbiol..

[233] Goli H.R., Nahaei M.R., Rezaee M.A., Hasani A., Kafil H.S., Aghazadeh M., Nikbakht M., Khalili Y. (2018). Role of MexAB-OprM and MexXY-OprM efflux pumps and class 1 integrons in resistance to antibiotics in burn and Intensive Care Unit isolates of *Pseudomonas aeruginosa*. J. Infect. Public Health.

[234] Beggs G.A., Brennan R.G., Arshad M. (2019). MarR family proteins are important regulators of clinically relevant antibiotic resistance. Protein Sci..

[235] Varela M., Kumar S. (2013). Molecular mechanisms of bacterial resistance to antimicrobial agents. Chemotherapy.

[236] Drawz S.M., Bonomo R.A. (2010). Three Decades of β-Lactamase Inhibitors. Clin. Microbiol. Rev..

[237] Bonomo R.A. (2017). β-Lactamases: A Focus on Current Challenges. Cold Spring Harb. Perspect. Med..

[238] Bush K., Jacoby G.A. (2010). Updated Functional Classification of β-Lactamases. Antimicrob. Agents Chemother..

[239] Sawa T., Kooguchi K., Moriyama K. (2020). Molecular diversity of extended-spectrum β-lactamases and carbapenemases, and antimicrobial resistance. J. Intensiv. Care.

[240] Bradford P.A. (2001). Extended-Spectrum β-Lactamases in the 21st Century: Characterization, Epidemiology, and Detection of This Important Resistance Threat. Clin. Microbiol. Rev..

[241] Yong D., Lee K., Yum J.H., Shin H.B., Rossolini G.M., Chong Y. (2002). Imipenem-EDTA Disk Method for Differentiation of Metallo-β-Lactamase-Producing Clinical Isolates of *Pseudomonas* spp. and *Acinetobacter* spp.. J. Clin. Microbiol..

[242] Jacoby G.A. (2009). AmpC β-Lactamases. Clin. Microbiol. Rev..

[243] Bush K., Jacoby G.A., Medeiros A.A. (1995). A functional classification scheme for beta-lactamases and its correlation with molecular structure. Antimicrob. Agents Chemother..

[244] Heo Y.A. (2021). Imipenem/Cilastatin/Relebactam: A Review in Gram-Negative Bacterial Infections. Drugs.

[245] Golkar T., Zieliński M., Berghuis A.M. (2018). Look and Outlook on Enzyme-Mediated Macrolide Resistance. Front. Microbiol..

[246] Zieliński M., Park J., Sleno B., Berghuis A.M. (2021). Structural and functional insights into esterase-mediated macrolide resistance. Nat. Commun..

[247] Xing L., Yu H., Qi J., Jiang P., Sun B., Cui J., Ou C., Chang W., Hu Q. (2015). ErmF and ereD Are Responsible for Erythromycin Resistance in Riemerella anatipestifer. PLoS ONE.

[248] Ramirez M.S., Tolmasky M.E. (2010). Aminoglycoside modifying enzymes. Drug Resist. Updat..

[249] Jaimee G., Halami P.M. (2015). Emerging resistance to aminoglycosides in lactic acid bacteria of food origin—An impending menace. Appl. Microbiol. Biotechnol..

[250] Disney M.D. (2011). Studying Modification of Aminoglycoside Antibiotics by Resistance-Causing Enzymes via Microarray. Breast Cancer.

[251] Jana S., Deb J.K. (2006). Molecular understanding of aminoglycoside action and resistance. Appl. Microbiol. Biotechnol..

[252] Labby K.J., Garneau-Tsodikova S. (2013). Strategies to overcome the action of aminoglycoside-modifying enzymes for treating resistant bacterial infections. Futur. Med. Chem..

[253] Gonzalez L.S., Spencer J.P. (1998). Aminoglycosides: A practical review. Am. Fam. Physician.

[254] Yang W., Moore I.F., Koteva K.P., Bareich D.C., Hughes D.W., Wright G.D. (2004). TetX Is a Flavin-dependent Monooxygenase Conferring Resistance to Tetracycline Antibiotics. J. Biol. Chem..

[255] Markley J.L., Wencewicz T.A. (2018). Tetracycline-Inactivating Enzymes. Front. Microbiol..

[256] Ye J., Chen X. (2022). Current Promising Strategies against Antibiotic-Resistant Bacterial Infections. Antibiotics.

[257] Isaev A.B., Musharova O.S., Severinov K.V. (2021). Microbial Arsenal of Antiviral Defenses—Part I. Biochemistry.

[258] Ryan E.M., Alkawareek M.Y., Donnelly R.F., Gilmore B.F. (2012). Synergistic phage-antibiotic combinations for the control of *Escherichia coli* biofilms in vitro. FEMS Immunol. Med. Microbiol..

[259] Comeau A.M., Tétart F., Trojet S.N., Prère M.-F., Krisch H.M. (2007). Phage-Antibiotic Synergy (PAS): β-Lactam and Quinolone Antibiotics Stimulate Virulent Phage Growth. PLoS ONE.

[260] Tagliaferri T.L., Jansen M., Horz H.-P. (2019). Fighting Pathogenic Bacteria on Two Fronts: Phages and Antibiotics as Combined Strategy. Front. Cell. Infect. Microbiol..

[261] Yacoby I., Shamis M., Bar H., Shabat D., Benhar I. (2006). Targeting Antibacterial Agents by Using Drug-Carrying Filamentous Bacteriophages. Antimicrob. Agents Chemother..

[262] Vaks L., Benhar I. (2011). In vivo characteristics of targeted drug-carrying filamentous bacteriophage nanomedicines. J. Nanobiotechnol..

[263] Panwar R.B., Sequeira R.P., Clarke T.B. (2021). Microbiota-mediated protection against antibiotic-resistant pathogens. Genes Immun..

[264] Hou K., Wu Z.X., Chen X.Y., Wang J.Q., Zhang D., Xiao C., Zhu D., Koya J.B., Wei L., Li J. (2022). Microbiota in health and diseases. Signal. Transduct. Target.

[265] Matzaras R., Nikopoulou A., Protonotariou E., Christaki E. (2022). Gut Microbiota Modulation and Prevention of Dysbiosis as an Alternative Approach to Antimicrobial Resistance: A Narrative Review. Yale J. Biol. Med..

[266] Garcia-Gutierrez E., Mayer M.J., Cotter P.D., Narbad A. (2018). Gut microbiota as a source of novel antimicrobials. Gut Microbes.

[267] Forslund K., Sunagawa S., Kultima J.R., Mende D.R., Arumugam M., Typas A., Bork P. (2013). Country-specific antibiotic use practices impact the human gut resistome. Genome Res..

[268] Hu Y., Yang X., Qin J., Lu N., Cheng G., Wu N., Pan Y., Li J., Zhu L., Wang X. (2013). Metagenome-wide analysis of antibiotic resistance genes in a large cohort of human gut microbiota. Nat. Commun..

[269] Stracy M., Snitser O., Yelin I., Amer Y., Parizade M., Katz R., Rimler G., Wolf T., Herzel E., Koren G. (2022). Minimizing treatment-induced emergence of antibiotic resistance in bacterial infections. Science.

[270] Zipperer A., Konnerth M.C., Laux C., Berscheid A., Janek D., Weidenmaier C., Burian M., Schilling N.A., Slavetinsky C., Marschal M. (2016). Human commensals producing a novel antibiotic impair pathogen colonization. Nature.

[271] Chakraborty N., Jha D., Roy I., Kumar P., Gaurav S.S., Marimuthu K., Ng O.-T., Lakshminarayanan R., Verma N.K., Gautam H.K. (2022). Nanobiotics against antimicrobial resistance: Harnessing the power of nanoscale materials and technologies. J. Nanobiotechnol..

[272] Rosini R., Nicchi S., Pizza M., Rappuoli R. (2020). Vaccines Against Antimicrobial Resistance. Front. Immunol..

[273] Wu J.Y., Srinivas P., Pogue J.M. (2020). Cefiderocol: A Novel Agent for the Management of Multidrug-Resistant Gram-Negative Organisms. Infect. Dis. Ther..

[274] Karlowsky J.A., Steenbergen J., Zhanel G.G. (2019). Microbiology and Preclinical Review of Omadacycline. Clin. Infect. Dis..

[275] Cohen D.T., Zhang C., Fadzen C.M., Mijalis A.J., Hie L., Johnson K.D., Shriver Z., Plante O., Miller S.J., Buchwald S.L. (2018). A chemoselective strategy for late-stage functionalization of complex small molecules with polypeptides and proteins. Nat. Chem..

[276] Newman D. (2022). Old and Modern Antibiotic Structures with Potential for Today’s Infections. ADMET DMPK.

[277] Flint A.J., Davis A.P. (2022). Vancomycin mimicry: Towards new supramolecular antibiotics. Org. Biomol. Chem..

[278] Figazzolo C., Bonhomme F., Saidjalolov S., Ethève-Quelquejeu M., Hollenstein M. (2022). Enzymatic Synthesis of Vancomycin-Modified DNA. Molecules.

[279] Yang J.H., Wright S.N., Hamblin M., McCloskey D., Alcantar M.A., Schrübbers L., Lopatkin A.J., Satish S., Nili A., Palsson B.O. (2019). A White-Box Machine Learning Approach for Revealing Antibiotic Mechanisms of Action. Cell.

[280] Aksamit T., Wu J., Hassan M., Achter E., Chatterjee A. (2022). Impact of initiation of amikacin liposome inhalation suspension on hospitalizations and other healthcare resource utilization measures: A retrospective cohort study in real-world settings. BMC Pulm. Med..

[281] https://www.fda.gov/news-events/press-announcements/fda-approves-new-antibacterial-drug-treat-serious-lung-disease-using-novel-pathway-spur-innovation.

[282] Cipolla D., Blanchard J., Gonda I. (2016). Development of Liposomal Ciprofloxacin to Treat Lung Infections. Pharmaceutics.

[283] Micoli F., Bagnoli F., Rappuoli R., Serruto D. (2021). The role of vaccines in combatting antimicrobial resistance. Nat. Rev. Genet..

[284] Huang W., Zhang Q., Li W., Yuan M., Zhou J., Hua L., Chen Y., Ye C., Ma Y. (2019). Development of novel nanoantibiotics using an outer membrane vesicle-based drug efflux mechanism. J. Control. Release.

[285] Lee N.-Y., Ko W.-C., Hsueh P.-R. (2019). Nanoparticles in the Treatment of Infections Caused by Multidrug-Resistant Organisms. Front. Pharmacol..

[286] Galbadage T., Liu D., Alemany L.B., Pal R., Tour J.M., Gunasekera R.S., Cirillo J.D. (2019). Molecular Nanomachines Disrupt Bacterial Cell Wall, Increasing Sensitivity of Extensively Drug-Resistant Klebsiella pneumoniae to Meropenem. ACS Nano.

[287] Zhou Y., Deng W., Mo M., Luo D., Liu H., Jiang Y., Chen W., Xu C. (2021). Stimuli-Responsive Nanoplatform-Assisted Photodynamic Therapy Against Bacterial Infections. Front. Med..

[288] Shih Y.-H., Yu C.-C., Chang K.-C., Tseng Y.-H., Li P.-J., Hsia S.-M., Chiu K.-C., Shieh T.-M. (2022). Synergistic Effect of Combination of a Temoporfin-Based Photodynamic Therapy with Potassium Iodide or Antibacterial Agents on Oral Disease Pathogens In Vitro. Pharmaceuticals.

[289] Bouley R., Kumarasiri M., Peng Z., Otero L.H., Song W., Suckow M.A., Schroeder V.A., Wolter W.R., Lastochkin E., Antunes N.T. (2015). Discovery of Antibiotic (*E*)-3-(3-Carboxyphenyl)-2-(4-cyanostyryl)quinazolin-4(3*H*)-one. J. Am. Chem. Soc..

[290] Janardhanan J., Bouley R., Martínez-Caballero S., Peng Z., Batuecas-Mordillo M., Meisel J.E., Ding D., Schroeder V.A., Wolter W.R., Mahasenan K.V. (2019). The Quinazolinone Allosteric Inhibitor of PBP 2a Synergizes with Piperacillin and Tazobactam against Methicillin-Resistant Staphylococcus aureus. Antimicrob. Agents Chemother..

[291] Perry J., Waglechner N., Wright G. (2016). The Prehistory of Antibiotic Resistance. Cold Spring Harb. Perspect. Med..

[292] Dadgostar P. (2019). Antimicrobial Resistance: Implications and Costs. Infect. Drug Resist..

[293] Plackett B. (2020). Why big pharma has abandoned antibiotics. Nature.

[294] Kållberg C., Blix H.S., Laxminarayan R. (2019). Challenges in Antibiotic R&D Calling for a Global Strategy Considering Both Short- and Long-Term Solutions. ACS Infect. Dis..

[295] Klug D.M., Idiris F.I.M., Blaskovich M.A.T., von Delft F., Dowson C.G., Kirchhelle C., Roberts A.P., Singer A.C., Todd M.H. (2021). There is no market for new antibiotics: This allows an open approach to research and development. Wellcome Open Res..

[296] https://www.amractionfund.com.

